# Systematic Data-Driven Modeling of Bimetallic Catalyst Performance for the Hydrogenation of 5-Ethoxymethylfurfural with Variable Selection and Regularization

**DOI:** 10.1021/acs.iecr.1c03995

**Published:** 2022-03-31

**Authors:** Pekka Uusitalo, Aki Sorsa, Fernando Russo Abegão, Markku Ohenoja, Mika Ruusunen

**Affiliations:** † Environmental and Chemical Engineering Research Unit, Control Engineering Group, Faculty of Technology, P.O. Box 4300, University of Oulu, Oulu 90014, Finland; ‡ School of Engineering, Newcastle University, Newcastle upon Tyne NE1 7RU, United Kingdom

## Abstract

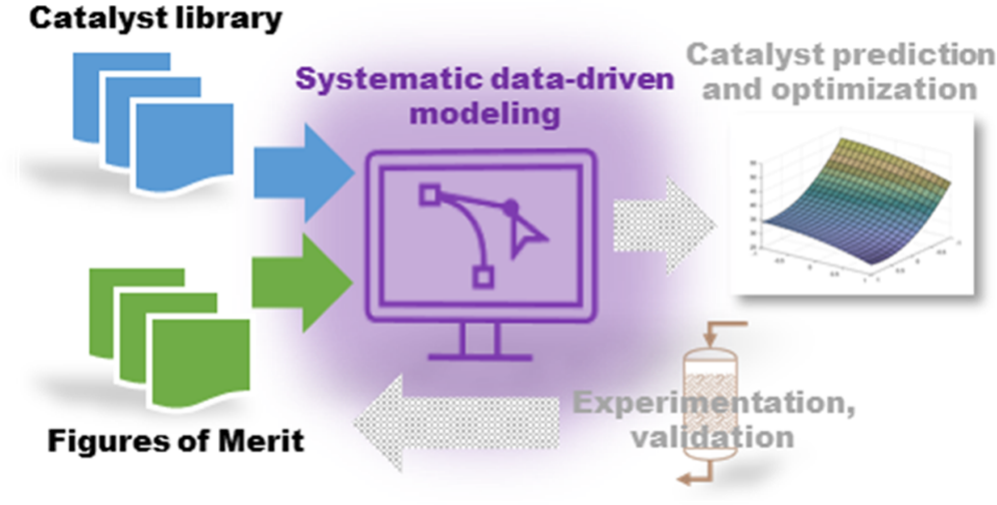

Catalyst development for biorefining applications involves many challenges. Mathematical modeling can be seen as an essential tool in assisting to explain catalyst performance. This paper presents studies on several machine learning (ML) methods that can model the performance of heterogeneous catalysts with relevant descriptors. A systematic approach for selecting the most appropriate ML method is taken with focus on the variable selection. Regularization algorithms were applied to variable selection. Several different candidate model structures were compared in modeling with interpretation of results. The systematic modeling approach presented aims to highlight the necessary tools and aspects to unexperienced users of ML. Literature datasets for the hydrogenation of 5-ethoxymethylfurfural with simple bimetal catalysts, including main metals and promoters, were studied with the addition of catalyst descriptors found in the literature. Good results were obtained with the best models for estimating conversion, selectivity, and yield with correlations between 0.90 and 0.98. The best identified model structures were support vector regression, Gaussian process regression, and decision tree methods. In general, the use of variable selection procedures was found to improve the performance of models. The modeling methods applied thus seem to exhibit a strong potential in aiding catalyst development based mainly on the information content of descriptor datasets.

## 1 Introduction

Replacing fossil-based fuels and products with bio-based counterparts produced from renewable resources is of great interest due to climate change and depleting fossil-based feedstocks. Fossil-based feedstocks consist of hydrocarbons, which makes catalysis relatively easy. In contrast, biomass has overfunctionalized composition, where alcohols, ethers, esters, and carboxylic acids are present, thus making the conversion of biomass challenging.^1^ Additionally, complex reaction mixtures including poorly characterized components are involved, and a large number of inorganic species in various concentrations are present, depending on the source and type of biomass.^2^ Catalysis for biorefining applications therefore involves many challenges due to its complex nature.^3−5^ It is important to design catalysts with appropriate structure and electronic properties to favor the chemistries of interest, it is necessary to ensure that particle morphology is appropriate to minimize mass transfer resistances, and it is important to ensure catalyst lifetime and integration with the reactor environment. Additionally, for biorefineries to thrive and be able to financially compete with fossil-based manufacturing and ensure sustainability of the processes, it is necessary to minimize the use of precious metals and metals from conflict areas. In contrast to traditional catalysis development approaches, the methodologies followed in BioSPRINT,^6^ an EU funded project, involve judicious design of catalyst formulations through exploration of wider experimental spaces combining multimetal chemistries to find out nontrivial synergies and enhanced formulations that will mostly rely on affordable and sustainable metals. The mathematical modeling approach presented in this study could help to explain the complex dependencies between catalyst formulation and performance. Hence, modeling can be an essential tool in catalyst development.^7,8^

Modeling in catalysis can be fundamental, empirical (data-driven), or a combination thereof.^8^ The empirical approach aims to find correlations between the catalyst descriptors, in other words computational features describing the physicochemical properties of the catalysts, and figures of merit (FOM), for example, product selectivity, yield, turnover number and frequency, cost per kg, or a combination of these.^9^ It is based on data without assumptions on reaction mechanisms and reactor configurations. Empirical models are based on statistical analysis and are often combined with stochastic optimization methods.^8,10^

According to Madaan et al.^8^ and Ras et al.,^11^ catalyst performance could be predicted using data-driven modeling with simple descriptors. These models can be less interpretable but more practical than methods that require high computational power and use complex algorithms with quantum and classical mechanics.^8^ However, Madaan et al.^8^ point out that both fundamental and empirical approaches are needed for finding new catalysts and optimizing the existing ones. The lack of universal databases for catalytic FOMs and descriptors hinders the use of data-driven methods in catalysis.^4^ Empirical approaches are fast, but these adapt poorly to new factors and interpretability can be weak.^8^ In combination of fundamental and empirical approaches, descriptors based on chemical principles can also be combined with statistical modeling.^8^ This approach is preferable for predicting catalyst performance.^8,10^ Examples of different modeling approaches are listed in Madaan et al.’s^8^ study.

Machine learning (ML) enhances ways to discover catalysts, generates knowledge about catalysis, and establishes a deeper understanding of the relationship between material properties and their catalytic FOMs.^3−5^ With combined computational modeling and/or experiments, catalysts can be rapidly screened, descriptors of catalyst performance can be found, and catalyst synthesis can be enhanced.^7^ ML can also be used in formulating new descriptors used in combination with quantum mechanical methods and to formulate interatomic potentials.^3,7^

The use of ML in computational catalysis research and integration with experimental research programs has been increasing.^3^ Goldsmith et al.^7^ listed several examples, where integration of ML and high-throughput (HTP) screening for heterogeneous catalysts was used to predict catalyst FOM for large catalyst spaces. However, it has been concluded that the predictions of catalytic FOM are still in their early stages.^7^ Synthesis conditions and compositions have been used as model input variables for predictive models, which can be seen, for example, in Baumes et al.^12,13^ These ML approaches can guide the synthesis toward better catalysts, although data from experiments are often incomplete and can result in poorly generalized models for large chemical spaces.^7^ Hence, usually multiple iterations and experimentations are required for the successful application of ML in catalyst development.^9^

The early work in applying ML with focus on homogeneous catalysis has been reviewed by Maldonado and Rothenberg.^9^ The same main principles can be applied for heterogeneous catalysis too. From the ML point of view, the model structures encountered in the published studies have involved, for example, genetic algorithms (GAs) and artificial neural networks (ANNs),^14,15^ Gaussian process regression (GPR), radial basis function network (RBFN), support vector regression (SVR),^16^ orthogonal partial least squares (OPLS),^11^ and random forest (ensemble of decision trees).^17,18^ In comparison, modeling methods used in this study are linear regression, decision tree regression, SVR, ensemble tree regression, and GPR. Also, partial least squares regression (PLSR) and regularization regression are used to identify reference models.

Despite the fact that several studies can be found where ML is used in catalysis, only in a few studies, a systematic approach (involving data preprocessing, variable selection, modeling, and validation steps) for selecting the ML method is taken. In addition, the variable selection step has not gained significant focus in most of the earlier works, although it is an important step in identifying low-dimensional ML models applicable on small datasets.

Earlier research has proved that it is possible to describe catalyst performance well with mathematical models developed via variable selection. In the study of Procelewska et al.,^19^ different variable selection methods were tested to find the relevant descriptors for predicting solid catalyst performance in the propene oxidation reaction. Also, Goldsmith et al.^7^ listed some references, where variable selection was studied; in particular, the use of the sure independence screening and sparsifying operator (SISSO)^20^ algorithm was mentioned. In both of these studies, data-based variable selection was found to be a promising method to find important variables from a high-dimensional feature space, while reducing the dimensionality of the identified models. In a similar manner, in this study, regularization algorithms are used as variable selection methods. Researchers have used regularized algorithms to study catalysis, such as Lasso.^21,22^

In this paper, a systematic approach for testing and comparing different ML approaches with variable selection is taken. The aim in this study is to identify a model structure that is as incomplex as possible but still able to model the outputs accurately. A variable selection procedure is applied to find a subset of relevant input variables and thus make the model structure simpler and to avoid overfitting problems often encountered with small datasets. Different ML modeling approaches are tested to increase knowledge of the suitability of these methods to the task. The systematic approach implemented with readily available and easily approachable methods also aims to give an insightful example of the utilization of ML to chemical engineers with limited experience in ML.

The reaction studied in this work is the hydrogenation of 5-ethoxymethylfurfural (5-EMF), which is an important reaction related to the conversion of biomass into biofuels.^1,23,24^ The desired product is 5-ethoxymethylfurfuryl alcohol, which is a potential additive for diesel fuel.^1^ 5-EMF can be easily obtained via acid-catalyzed dehydration of C6 sugars with ethanol as a solvent.^1^ According to the best knowledge of the authors, the studies of Ras et al.^1,11,25^ are the only openly available studies that focus specifically on this reaction with the aim of modeling the catalyst performance. Studies that focus on the synthesis of 5-EMF can be found, for example, in refs (23, 24). This article focuses on ML and modeling rather than on the chemistry of the reaction. References to studies where chemistry is more thoroughly studied can be found, for example, in refs (1, 23, 24).

This article is structured as follows: Section 2 presents the considered systematic approach, datasets and methods for modeling, and variable selection. This is followed by the results and discussion in Section 3, where modeling with and without variable selection is studied, the models are compared to the models found in similar studies, model residuals are analyzed, and the variable importance is analyzed by studying the correlations and variable occurrences for each response. Finally, Section 4 concludes the findings of this study.

## 2 Materials and Methods

**Figure 1:**
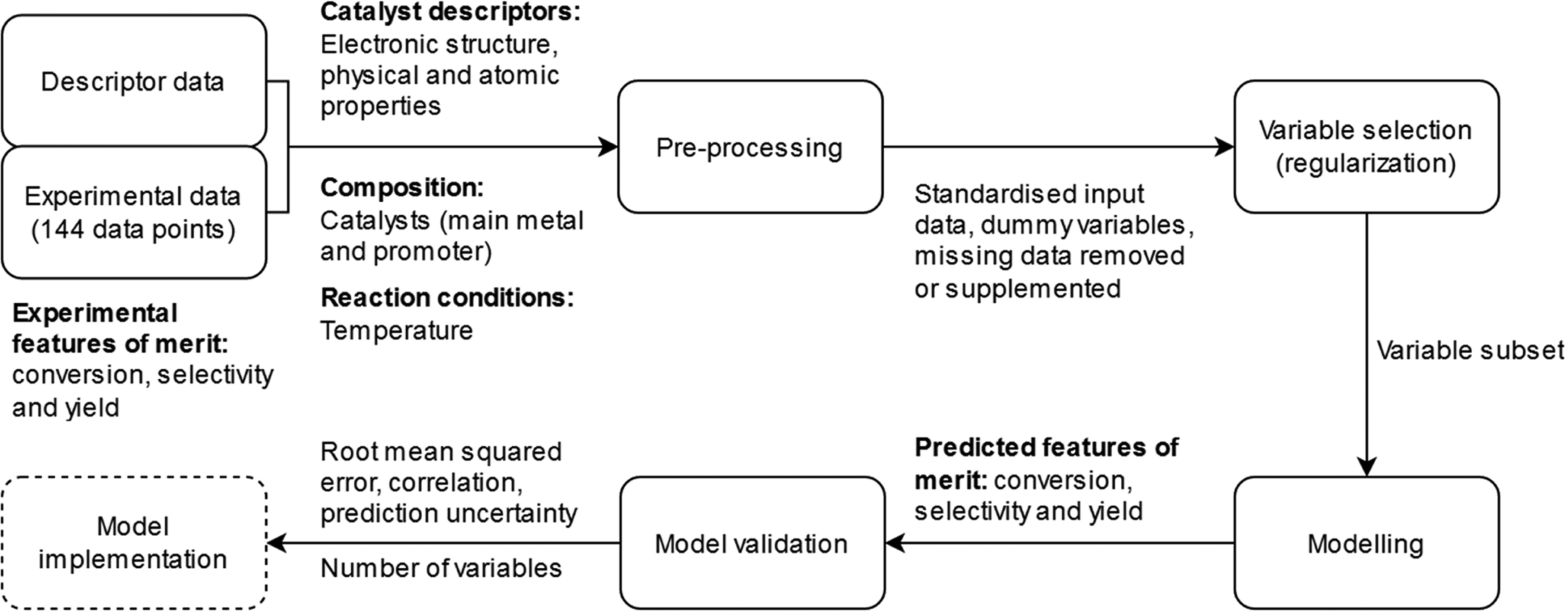
Systematic modeling approach for this study.

The systematic model identification approach of this study is illustrated in Figure 1. First, a catalyst library, in this case from the literature, was obtained with catalyst compositions. Second, a dataset for catalyst descriptors was obtained from the literature. These descriptors included electronic structure, physical, and atomic properties. Also, reaction conditions, namely, measured temperatures during experiments were included. The obtained data were then preprocessed, where data were standardized, variables with missing values were removed, and categorical variables were converted into dummy variables. With the preprocessed data, variable selection was then performed by regularization algorithms. The obtained variable subset was then used with the model structures found with the Regression Learner App (RLA) in MATLAB. With the obtained FOM predictions, the model performance was finally evaluated. The identified models could then be implemented to the catalyst development framework to aid the experimental designs and catalyst synthesis.

### 2.1 Datasets

The experimental data as published by Ras et al.^1^ was utilized. The dataset consists of conversion and selectivity as responses, namely, FOMs. Also, their product, namely, yield, was selected in this study as response. Literature data were available from multiple experiments, with 48 different catalyst combinations in three different temperatures (80, 100, and 120 °C) and with two solvents (diethyl carbonate and 1,4-dioxane). Eight different main metals (Au, Cu, Ir, Ni, Pd, Pt, Rh, Ru) and six promoters (Bi, Cr, Fe, Na, Sn, W) had been used as catalysts. Al_2_O_3_ support had been applied in all observations. According to the original paper by Ras et al.,^1^ the main metal had a loading of 1 wt% and the promoter loading was 10 mol% in relation to the main metal. Feedstock composition remained constant in the studied dataset. In contrast to the approach by Ras et al.,^11^ where yield is predicted, also conversion and selectivity with two solvents were predicted separately in this study, to evaluate the most important descriptors for them. This led to six different models having each an individual response variable: conversion with diethyl carbonate solvent (C1), selectivity with diethyl carbonate solvent (S1), conversion with 1,4-dioxane solvent (C2), selectivity with 1,4-dioxane solvent (S2), yield with diethyl carbonate solvent (Y1), and yield with 1,4-dioxane solvent (Y2).

Slater orbitals (STO)^26^ were calculated according to the methodology presented by Ras et al.^11^ Calculations resulted in four variables to describe the wave function of the valence electrons in each metal: rAPEX, R(r)APEX, FWHH, and SKEW. Ras et al.^11^ described these variables in the following way: rAPEX is defined as the “distance of maximum probability of encountering a valence electron”, R(r)APEX is the “maximum value of the probability distribution”, FWHH is the “width of the probability distribution at the half-height”, and SKEW is the “measure for the asymmetry of the distribution”. Interaction and quadratic terms for the STOs were calculated and added to the descriptor data as well. In addition, descriptors based on the periodic table were added to the dataset.^27−30^ The database consisted thus 61 input variables and six response variables at this stage. The analyzed data are available in the Supporting Information.

### 2.2 Preprocessing

**Figure 2:**
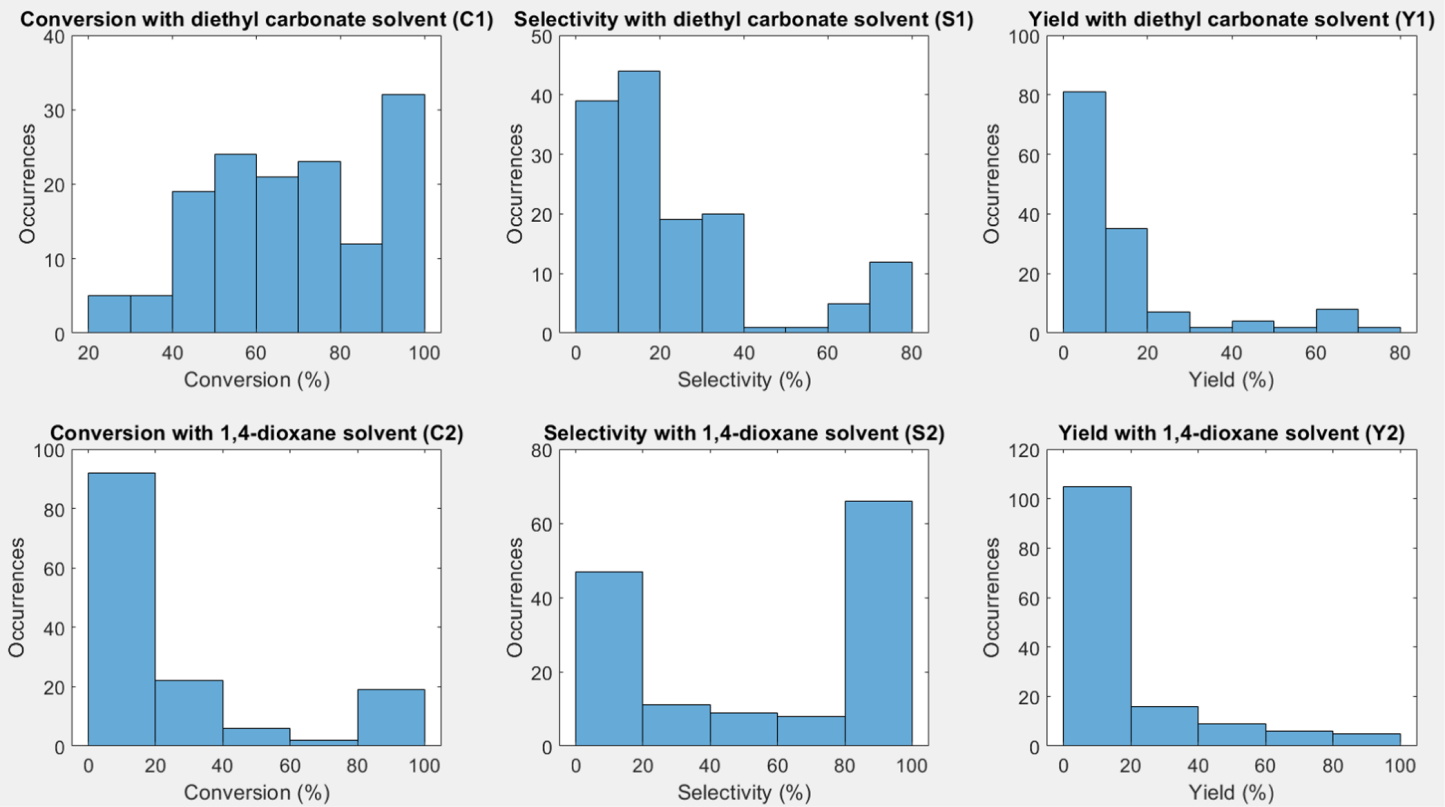
Histograms for each response describing the structures of experimental datasets. The *y*-axis shows the occurrences of each bin. The *x*-axis presents the conversion, selectivity, and yield values.

Based on the exploratory analysis of the experimental dataset (see Figure 2), it is notable that with 1,4-dioxane as a solvent, the dataset is poor for modeling of conversion (C2) and yield (Y2) since most of the observations are near zero and selectivity values (S2) are most often either near 0 or near 100. Also, yield with diethyl carbonate solvent (Y1) has most of the observed values near zero. This leads to response variables that remain constant even though there is variability in input variables. Correlation coefficients were further calculated between input variables and between input and output variables. The main results can be seen in Section 3.5.

Ir/W observations were unavailable for the first solvent, and because of that, these were removed in the preprocessing stage. Variables with missing values were also removed leading to 57 variables in total. Categorical variables were converted into dummy variables so that these were able to be utilized as input variables with the applied methods. A dummy variable is created for each category of each categorical variable. For example, there are eight different main metals. Accordingly, the same number of dummy variables were determined for categorical variable “main metal”. Each dummy variable defines each category with binary values 0 or 1: value 1 means true, and value 0 is false (for example, if the dummy variable for Au is 1, the main metal used is Au). Separate variables were obtained for main metals and promoters. The final dataset consisted of 147 variables after preprocessing (see Tables S15–S19 for a full list of used descriptors). Also, continuous variables were standardized so that each variable was set to mean value of 0 and standard deviation 1.

### 2.3 Regularization and Variable Selection

Variable subset selection can be divided into filters wrappers and embedded methods.^31^ In filtering, variables are ranked based on some criterion, which usually is the correlation coefficient. A subset of variables is selected independently of the chosen predictor as a preprocessing step. Wrapper methods select subset of variables based on their usefulness to a given predictor. There, a selected model structure is utilized with different subsets of variables to score them according to resulted modeling accuracy. Embedded methods resemble wrapper methods, but in addition to optimizing goodness-of-fit-term, they also penalize a large number of variables. Variable selection is performed during the training phase and is usually specific to the applied model structure.^31^

Regularization algorithms resemble embedded variable selection methods. Regularization aims to reduce the overfitting of the models. In this study, lasso and ridge regression and their combination, elastic net,^32−35^ were applied in the variable selection. With these methods, a regularization term is used in the model parameter identification step to decrease the parameter values and to avoid overfitting issues. Generally, variables with higher predictive power tend to gain larger coefficient values. Variables with coefficient values equaling to zero or below a threshold value were removed in the variable selection step. The methods for this study were limited to these due to the general use of lasso, ridge, and elastic net regularization in the field of ML and their ease of applicability. Variable subsets were also chosen manually based on experts’ knowledge and statistical analysis. With these selected methods, regularization is then adjusted with a parameter λ. The higher the λ value is, the more regularization is applied. A suitable value for λ needs to be determined so that the model is as simple as possible without significant loss of information in data.

For ridge regression and elastic net, thresholds for coefficient values were identified to limit the number of variables selected into the subset. Variables with higher coefficient values, namely, more relevant than the threshold value were kept in the subset. Thresholds were adjusted heuristically so that the gained variable subsets were kept at a reasonable size, therefore efficiently reducing the model’s complexity. For the lasso regularization, the variable selection is considered easier because the algorithm automatically typically sets most of the coefficient values to zero.^34^

### 2.4 Modeling Methods

In this study, decision trees, ensemble trees, support vector regression (SVR), and Gaussian process regression (GPR) were used in the modeling. These methods are introduced, for example, in the works of Witten, Mitchell, and Williams.^36−38^ In addition, partial least squares regression, linear regression, and regularized linear regression models were identified as reference methods. The implementation of these methods in this study is described more in detail in Section 2.7.

### 2.5 Data Division

The focus of this study was to study the interpolation capabilities of the models rather than extrapolation. The observations (equal to 141) were first split into training set (2/3 of the data) and test set (1/3 of the data) by performing a static data split. Stratified sampling was applied to ensure equal number of observations from each category (main metal, promoter, and temperature) in the test set. Detailed information on the data split can be found in the Supporting Information (see Tables S20–S23). Cross-validation (CV) was applied to model validation in the training phase. The CV subsets were used for selecting the relevant input variables and estimating the performance of the models with unseen data. Fivefold CV was used in both variable selection and modeling. The fivefold data split procedure was repeated with random resampling 94 (equal to the number of observations in the training set) times to avoid possible chance correlations.

### 2.6 Model Performance Metrics

The root mean square error (RMSE) values were calculated for the training data (denoted by RMSET, root mean square error of training), the CV data (denoted by RMSECV, root mean square error of cross-validation), and the test set (denoted by RMSEP, root mean square error of prediction) to validate models’ performance in this study. The prediction uncertainties (PUT = prediction uncertainty of training, PUCV = prediction uncertainty of cross-validation, and PUP = prediction uncertainty of prediction) were evaluated by calculating the mean value of error ± two times its standard deviation, and then indicating the interval, where approximately 95% of the errors are expected to be within this range. The Pearson correlation coefficient (RT = correlation of training, RCV = correlation of cross-validation, and RP = correlation of prediction, respectively) between measured and predicted values was also calculated to evaluate model performance.

A combination of Shapiro–Wilk and Shapiro–Francia tests^39^ was applied to test if the residuals follow normal distribution. An α value of 0.01 was set to the significance level. The results can be found in Section 3.4. The correlation between residuals and input variables was also calculated for the best models (see Section 3.4). Ideally, the correlation should be equal to zero. Thus, the variation in residuals is random.

### 2.7 Implementation of Computations in Software Environment

Preprocessing, variable selection, modeling, model validation, and statistical analysis were performed in this study with MATLAB. The fitrlinear function was used to perform variable selection with ridge and lasso regularization. A vector for different λ values was created, and the λ value that minimizes the mean square error (MSE) was chosen. The fitrlinear function with CV trains a model for each fold. Therefore, the variables chosen were determined for each model (in this case, 5). After this, variables that occurred at least in half of the folds (in this case, 3 or more) were chosen into the final subset. This procedure was executed 20 times, and the variables that occurred in half of the iterations were chosen in the final subset. This was done to minimize the amount of randomness due to CV, while keeping the computation times relatively short. Variable selection with fitrlinear function and ridge regression was performed with Stochastic Gradient Descent solver. With lasso, in contrast, Sparse Reconstruction by Separable Approximation was used. The least squares learner was used for both methods. MATLAB algorithms are described more in detail in the MATLAB documentation.

The lasso function was also used in MATLAB to perform lasso variable selection and elastic net variable selection. Elastic net variable selection was performed with an α value of 0.5. For both methods, variable selection was performed with two different λ values: a λ value that gives the minimum mean square error value (minMSE) and a λ value that is the largest λ value, one standard error away from the minMSE λ value (1SE). Thus, the 1SE λ value will give a smaller variable subset with a slightly larger MSE value.

The Statistics and Machine Learning Toolbox in MATLAB was used, which includes the Regression Learner App (RLA). All model structures found in the RLA were used in the study with some exceptions; stepwise linear regression was excluded because variable selection was carried out with embedded methods. In addition, coarse Gaussian SVR gave poor results, and the calculation times were long. Hence, the results with this method were also omitted. Variable subsets had to be restricted in some cases; the interactions linear model was only used with variable subsets smaller than 30 due to the increasing number of interaction terms. With linear, quadratic, and cubic SVR models, the kernel scale value was set to 1 instead of an automatically chosen value. This was done because with automatically chosen kernel scale values, the calculation times were longer, and in some cases, the RMSE values were extremely high. The applied methods included linear regression, decision trees, SVR, ensemble trees, and GPR. In addition, PLSR (which is commonly seen in ML-oriented catalyst model development) and linear regression models with regularization were identified as reference model structures. Finally, the modeling was performed with candidate subsets selected by the variable selection methods (Section 2.3) and to some extent with heuristically chosen variables (Table S8, variable subset VII). The readily available MATLAB functions for the RLA models include different ways to tackle overfitting (regularization), which differs for each modeling approach. However, this paper focuses on the regularization used in the variable selection. More detailed information about the possibilities with the various model structures can be found from the MATLAB documentation and introductory ML studies.^36,37^

## 3 Results and Discussion

This section is structured as follows: Section 3.1 presents the modeling results with reference models without variable selection. In Section 3.2, the modeling results with variable selection are presented. The results are compared to the reference models. Also, scatter plots of the best models can be found, where clear outliers are identified. This is followed by Section 3.3, where typical model performance of similar studies found in the literature is compared to the results of this study. Also, the models’ complexity is discussed. In Section 3.4, the model residuals are analyzed to point out possible problems in the error criteria. Eventually, the modeling is based on minimizing one of these. The residual distributions are tested to see if they follow normal distribution. In addition, the randomness of the residuals is analyzed by comparing correlations of residuals to the considered input variables. In Section 3.5, the descriptors’ importance is analyzed by calculating the correlation coefficients between input and output variables and calculating the variable occurrences for each response with all of the variable selection methods.

### 3.1 Modeling Results without Variable Selection

First, reference models were identified without variable selection. In Tables S1–S6 (see the Supporting Information), the results of reference models in terms of model performance metrics (Section 2.6) are given for each response. Results obtained with the following models are included: PLSR, lasso and elastic net regression with lasso function, lasso and ridge regression with fitrlinear function.

In general, for the models in Tables S1–S6, the lasso function with lasso or elastic net regularization tends to work best, when considering RMSEP values. Consistently large modeling errors are obtained for the response S2. This can be explained due to its challenging data structure as already mentioned in Section 2.1: The data distribution is uneven and most of the observations are extreme values (for example, either 100 or 0). For the reference models, the RP value ranges are 0.77–0.78 for C1, 0.65–0.76 for S1, 0.85–0.87 for C2, 0.48–0.54 for S2, 0.57–0.73 for Y1, and 0.65–0.69 for Y2.

In Table S7, the best results for each response without variable selection can be seen (first rows for each response). Compared to the results in Tables S1–S6, it can be noticed that significantly better results are obtained with quadratic SVR, fine tree, and boosted ensemble tree methods identified with the RLA (RMSEP values improved 4.2% for C1, 9.5% for S1, 8.9% for C2, 6.6% with S2, 3.0% with Y1, and 4.7% with Y2, compared with the best reference model). This highlights the applicability of RLA methods over the basic/regular modeling approaches (PLSR, fitlinear, lasso). However, the models are highly complex without variable selection procedures.

### 3.2 Modeling with Variable Selection

After identification of the reference models, modeling was performed with variable selection. The best results for each response can be seen in Table S7. The used variable subsets are depicted in Table S8, and the definitions for all of the used variables can be found in Tables S15–S19. According to Table S7, the best results in general were obtained with GPR, cubic SVR, and fine tree models (best RMSEP value 9.5% for C1, 4.9% for S1, 4.1% for Y1, 9.1% for C2, 34.1% for S2, and 8.8% for Y2). Four different kernel functions (squared exponential, matern 5/2, exponential, and rational quadratic) were used for GPR, which all gave almost equal results for RMSEP. Only the best one was involved in Table S7 for each response. In most cases, the fitrlinear function with ridge regularization worked best in variable selection. As mentioned in Section 2.3, the size of the variable subset was adjusted by changing the threshold value for model parameters. Therefore, smaller subsets were achieved in comparison to lasso algorithms. The elastic net variable selection tended to choose dummy variables more often than the other methods. The performance of the identified models may improve further with the use of hyperparameter optimization.

In comparison to the reference results in Tables S1–S6, the use of variable selection methods and the models from RLA here seem to have a beneficial impact on the results. For the best models (excluding the best results without variable selection), the RP value ranges are 0.90–0.90 for C1, 0.97–0.97 for S1, 0.96–0.98 for Y1, 0.95–0.96 for C2, 0.65–0.66 for S2, and 0.84–0.94 for Y2. In general, significant improvement is obtained using variable selection methods in combination with the RLA models, which can be noticed by comparing the range of values of RMSEP, RP, and PUP between the reference models and the best models seen in Table S7. The best RMSEP values are obtained for the response C1 with the reference models. Poor results (RMSEP > 30%) were obtained for response S2 with all of the methods. Even though responses C2, Y1, and Y2 had also a challenging data structure, good (RMSEP = 9.1%, 4.1%, and 8.8%, respectively) results were still obtained (see Table S7). When comparing the results with and without variable selection, it can be seen that with quadratic SVR, fine tree and boosted ensemble tree models almost equally good (RMSEP changed in comparison to the best results in the following way: −0.1% for C1, +0.4% for S1, +1.0% for Y1, −0.0% for C2, +0.4% for S2, and +4.3% for Y2) results are obtained without variable selection except for response Y2. Hence, similar (or even slightly better) model performance can be obtained with a simpler model structure using the studied regularization methods in variable selection. The results obtained show that variable selection is an important step when building models for catalyst performance.

**Figure 3:**
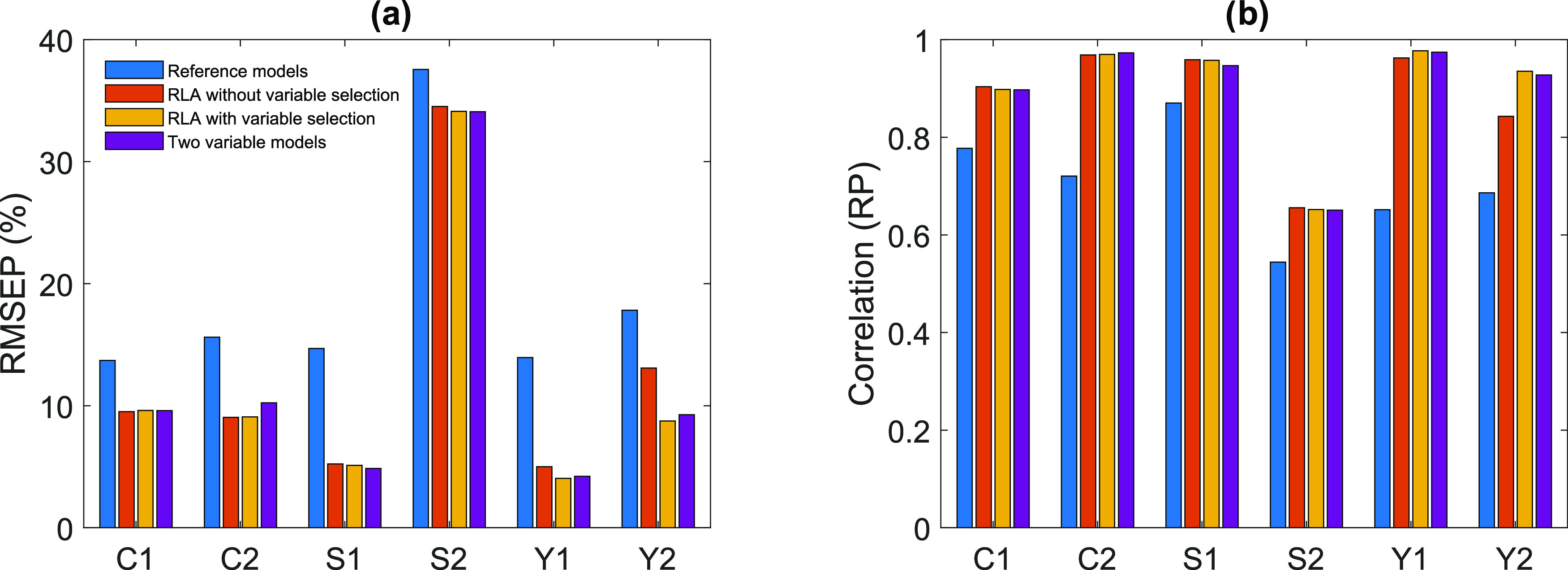
Best root mean square error of prediction (RMSEP) (a) and correlation (RP) (b) results for each response: conversion (C1), selectivity (S1), and yield (Y1) with diethyl carbonate solvent. Conversion (C2), selectivity (S2), and yield (Y2) with 1,4-dioxane solvent. RLA = regression learner app.

The best modeling results are summarized in Figure 3, including the best results for each response with reference models, RLA models without and with variable selection, and for the models with heuristically selected variables (temperature and Brinell hardness for main metal as inputs, see Section 3.5). As previously mentioned, the best results for S2 are insufficient (RMSEP > 34%). It can be noticed that in general, the best result with reference models (PLSR and regularization algorithms) are considerably worse than with the other methods. Overall, the RLA models without variable selection tend to have slightly worse results in comparison to the results with variable selection. In the case of response Y2, the results without variable selection are significantly worse. In general, the results with the heuristically selected variable models are only slightly worse than the models with the variable subsets chosen by the variable selection algorithms. As conclusion, the use of RLA models tends to improve the results in comparison to the reference models. The variable selection with regularization tends to improve the results by reducing the dimensionality of the models.

**Figure 4:**
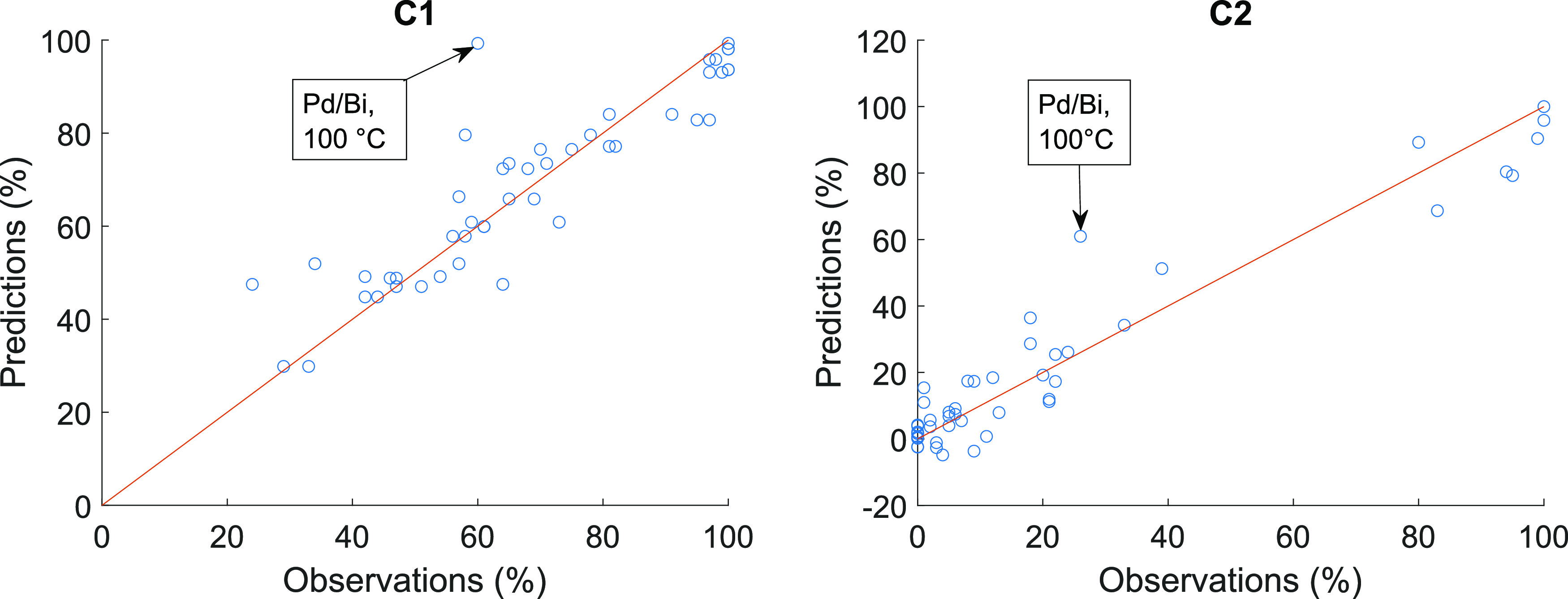
Predictions vs observations with the best models for predicting conversions with diethyl carbonate solvent (C1) (model no. 32) and with 1,4-dioxane solvent (C2) (model no. 41) with test set. RMSEP = 9.6%, RP = 0.90 for C1 and RMSEP = 9.1%, RP = 0.96 for C2.

**Figure 5:**
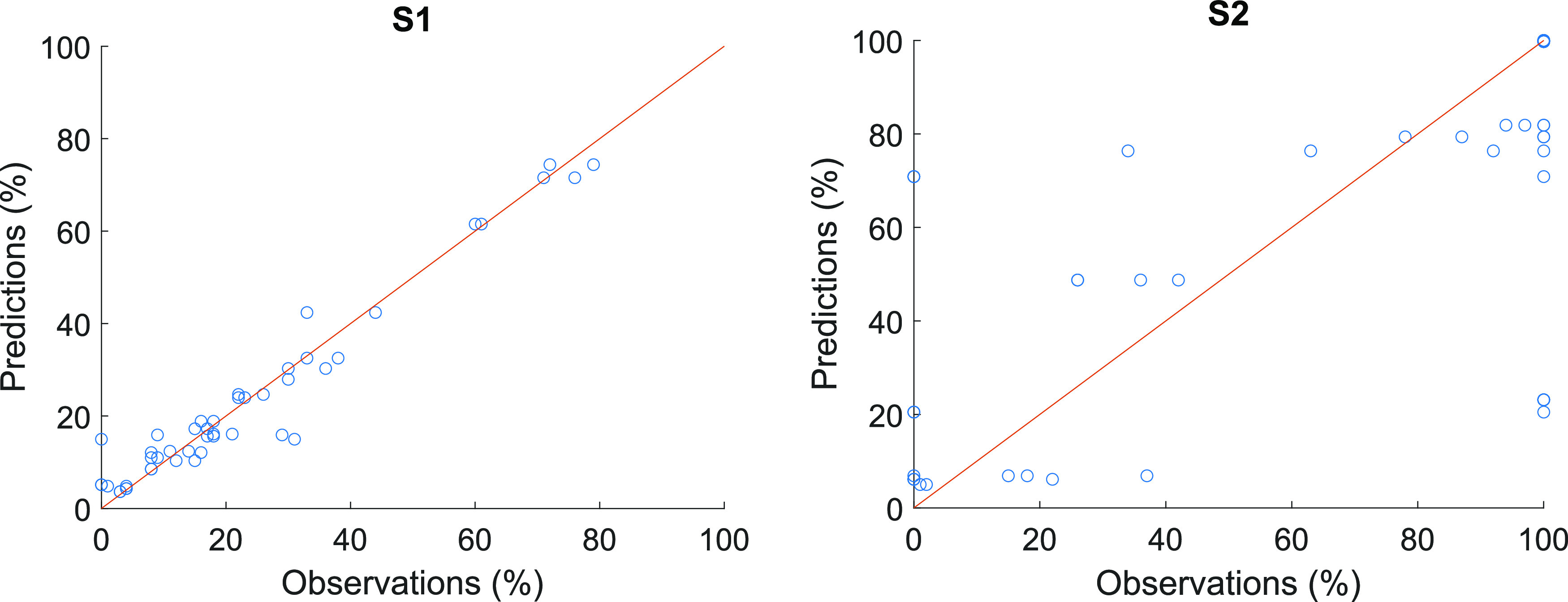
Predictions vs observations with the best models for predicting selectivities with diethyl carbonate solvent (S1) (model no. 36) and with 1,4-dioxane solvent (S2) (model no. 45) with test set. RMSEP = 4.9%, RP = 0.97 for S1 and RMSEP = 34.1%, RP = 0.65 for S2.

**Figure 6:**
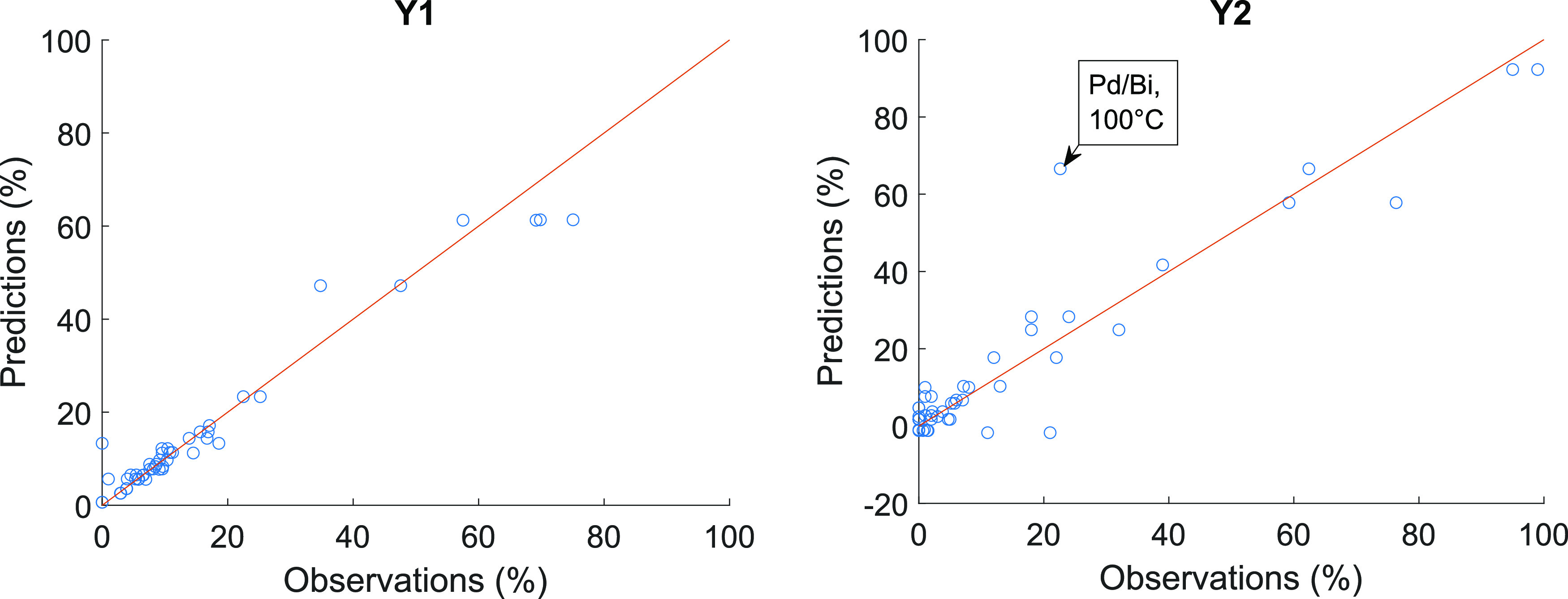
Predictions vs observations with the best models for predicting yields with diethyl carbonate solvent (Y1) (model no. 38) and with 1,4-dioxane solvent (Y2) (model no. 47) with test set. RMSEP = 4.1%, RP = 0.98 for Y1 and RMSEP = 8.8%, RP = 0.95 for Y2.

Scatter plots for each of the best test set predictions for each response can be seen in Figures 4–6. The figures support the conclusions made from the calculated error metrics. Outliers are marked in the plots except for response S2, whose results were poor (RMSEP > 30%). The outliers were detected visually. Later, in residual analysis (see Section 3.4), outliers are detected mathematically. From the predictions of C1, C2, and Y2 (see Figures 4 and 6), it can be noticed that Pd/Bi at temperature 100 °C is identified as an outlier. This can be expected since Pd follows a different reaction pathway according to Ras et al.^1^ More detailed information about the models can be found in the Supporting Information.

### 3.3 Qualitative Comparison to Other Research

Direct comparison of model performance metrics in data-driven modeling is challenging, as different datasets or data divisions are often used, and the modeling performance is inherently tied to the underlying data. The models identified in this study can be compared to the models proposed by Ras et al.^11^ However, there are some differences between the used experimental datasets and the descriptor datasets. Modeling results for predicting yield (product of conversion and selectivity) with the used experimental dataset can be found in the study of Ras et al.^11^ with one solvent (diethyl carbonate). In that same study, with bimetallic catalyst dataset, an *R*^2^ value equal to 0.79 (with the test set) was obtained by modeling the yield of unsaturated alcohol and diether together. The yield of the unsaturated alcohol followed by the hydrogenation of the carbonyl group was also predicted separately (*R*^2^ = 0.90 with the test set). In that study, only data corresponding to 80 °C temperature experiments were used. In addition to the bimetallic catalyst dataset results, modeling results for combined yield predictions (four reactions) with monometallic catalyst dataset can be found in the paper of Ras et al.^11^ with an *R*^2^ value equal to 0.76 (with test set). With the same catalyst dataset, the yield of the diether was predicted with training set (*R*^2^ = 0.80 without removing outliers and *R*^2^ = 0.91 after removing two outliers). It seems that similar results were obtained when comparing the best RP values in this study for responses C1, S1, C2, Y1, and Y2 (see Table S7) and the results for Ras et al.’s^11^ models.

The number of variables differs from the studied subsets. When evaluating the complexity of the models, it should be noted that the presence of continuous variables makes the model more complex than the presence of binary valued dummy variables. Thus, the number of variables in the studied subset cannot be solely considered. The number of input variables in the models seen in Table S7 (excluding the models, where all of the variables in the studied dataset were used) varies between 2 and 13. The models with only two input variables (subset XVII) performed well in comparison to more complex model structures. However, some of the studied models may be highly complex. The complexity could be further reduced by increasing the value of λ in the regularization part and/or by lowering the coefficient threshold value with ridge and elastic net regularization, with potential small accuracy loss in the model performance. In comparison, Ras et al.^10^ have used stepwise elimination of redundant variables to reduce the number of variables for a case with a monometallic dataset. Other studies in heterogeneous catalysis have also demonstrated that low-dimensional models can be achieved via the use of principal component analysis (PCA) and partial least squares (PLS).^8,40^

### 3.4 Residual Analysis

**Figure 7:**
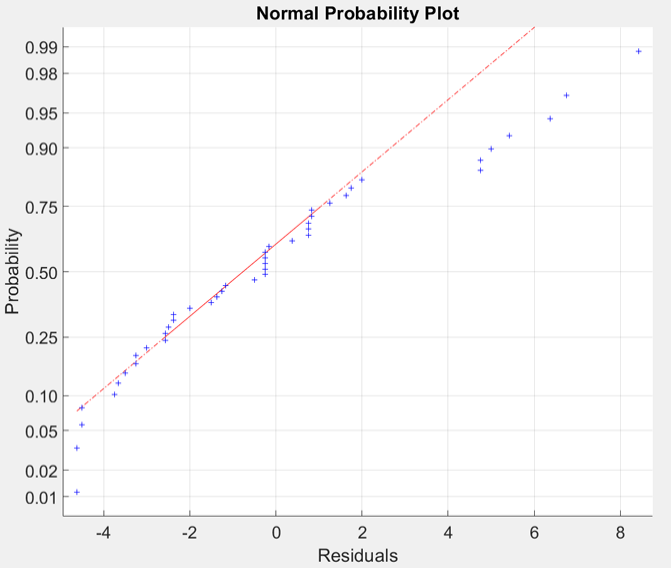
Normal probability plot of Fine Tree model’s residuals for test set after removing outliers for response S1 with ridge (fitrlinear) variable selection.

**Figure 8:**
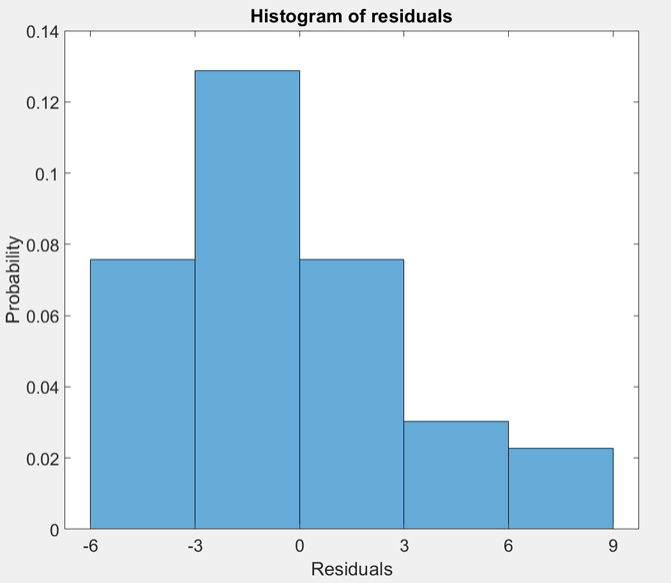
Histogram of residuals for test set after removing outliers for Fine Tree model for response S1 with ridge (fitrlinear) variable selection.

A combination of Shapiro–Wilk and Shapiro–Francia tests was used^39^ to test if the residuals of the best models for test set follow normal distribution. The Shapiro–Francia test was used with residual vectors where kurtosis was leptokurtic (kurtosis > 3). In other cases, the Shapiro–Wilk test was performed. An α value of 0.01 was used. The test was executed without removing the outliers and after removing outliers. Observations that differed more than 3 times the scaled mean absolute deviation (MAD) from the median were removed (see MATLAB documentation for function rmoutliers). It was noticed that after removing the outliers, more normal distributed residuals were identified according to the test. When performing the test after removing outliers, normal distributed residuals were found almost for all of the good models, when considering the calculated error metrics. Although some exceptions exist, for example, the Quadratic SVR model for response S1 had good metric values with variable selection method ridge (fitrlinear) but failed to produce normally distributed residuals according to the test. An example of the normal probability plot of Fine Tree model’s residuals for the test set for response S1 with ridge (fitrlinear) variable selection method can be seen in Figure 7. The residuals are normally distributed according to the Shapiro–Wilk test even though the data points outside the interquartile range (middle area between 75th and 25th percentiles) do not strictly follow the theoretical red line of normal distribution. A histogram for the same residuals can be seen in Figure 8.

The correlation between residuals and input variables was also calculated for the best models (models in Table S7). Correlations of models for response S2 were not analyzed. In general, low correlations (absolute correlation value < 0.25) were obtained for most of the variables with the studied methods. The highest correlations in general were obtained with the test set in comparison to the correlations with training set and CV set. The largest absolute correlation value (*R* = 0.30) with test set was obtained between the boiling point of the main metal and yield with diethyl carbonate solvent (Y1). Absolute correlation values between 0.25 and 0.35 were obtained for a few variables, when considering the correlations with training, CV, and test sets. Overall, the correlations were relatively low. In Section 3.5, the importance of descriptor will be discussed.

### 3.5 Descriptor Importance Analysis

Correlations between input variables and responses were calculated. Only the correlations for variables in the best models are considered here. Abbreviations M and P are used for main metal and promoter, respectively. Temperature seems to have the highest correlation in contrast to response S1 (*R* = −0.62), and also has a moderately high correlation value for Y1 (*R* = −0.51). Several STO variables with the addition of Brinell hardness and resistivity (Resistivity_M) for main metal have moderate correlation (|*R*| ≥ 0.40) for conversion responses (C1 and C2). Low correlation values (|*R*| ≤ 0.25) were obtained for both Brinell hardness for main metal and temperature in comparison to responses S1, Y1, and Y2 even though good results (RMSEP = 4.9, 4.2, and 9.3%, respectively) were obtained by only using these variables in the predictions.

Table S8 in the Supporting Information presents the variable subsets of some of the best models. It can be observed, for instance, that variables temperature and Brinell hardness for main metal are often seen among the selected variables. Next, the occurrences of variables (descriptors) are discussed in more detail. The abundance of each variable with different methods was evaluated with four different regularization methods:

- elastic net (EN) with lasso function with α value 0.5 and minMSE λ value,
- lasso with lasso function with minMSE λ value (L1),
- lasso with fitrlinear function (L2) and
- ridge with fitrlinear function (RR).

In contrast to the previous data division with separate test data, here, the whole dataset was used in combination with CV to evaluate the variable importance. Variable selection algorithms were executed in multiple iterations to minimize the effect of the randomness of the CV in the data division. Each algorithm was executed 20 times, resulting in a total number of 80 iterations. For the elastic net variable selection, the following threshold values (see Section 2.3) for coefficients were used: 2.3 for conversion C1, 0.9 for selectivity S1, 2.7 for conversion C2, 2.0 for selectivity S2, 0.5 for yield Y1, and 1.0 for yield Y2. For ridge regression, the following threshold values were used: 1.8 for conversion C1, 1.1 for selectivity S1, 4.5 for conversion C2, 3.0 for selectivity S2, 1.1 for yield Y1, and 2.2 for yield Y2.

In Tables S9–S14 (cf. Supporting Information), occurrences for the most often selected variables for each response are represented with four different regularization methods and their sum. As seen in Table S9, temperature and Brinell hardness (M) occur in every chosen subset with response C1. Temperature logically affects the reaction rates, and therefore, the conversion observed. On the other hand, the correlation with materials hardness is not directly obvious. However, hardness has been correlated through first principles with the microstructure and chemical bonding in the materials such as in refs (41−43). Both microstructure and bonding determine the ability of catalytic active sites to interact effectively with reactant molecules depending on the possibility to establish effective bonds between the metal sites and the reactants based on geometry and strength of intermolecular forces leading to effective activation of those in the course of the reaction. Therefore, it is interesting to note that hardness can effectively summarize the effect of fundamental properties of the metallic elements, both in terms of mechanical behavior and catalytic behavior. Also, a strong STO interaction term (for RAPEX and FWHH (M)) is present, once again indicating the importance of the metal electronic structure in its catalytic behavior. Only one promoter variable (second lattice angle (P)) is present, which mainly describes the presence of Bi because the value is constant with every other promoter. Thus, it suggests that the presence of promoter Bi effects the outcome significantly.

In contrast to the impact on C1, it can be noticed from Table S10 that four promoter variables (dummy variable for Fe (P), dummy variable for group 8 in the periodic table (P), volume magnetic susceptibility (P), and electrical conductivity (P)) are present for response S1. This somehow indicates that promoters are more likely to affect selectivity than conversion.

Slater interaction and quadratic terms are in the absence of response S1. Temperature, boiling point (M), and bulk modulus (M) occur in every studied subset. Electron affinity (M) and density (M) seem to be also important variables in modeling. This finding suggests that for this reaction, selectivity is affected by molecular-level electronic interactions with the catalyst more than by its structure and chemical bonding.

Similar trends were observed from Table S11. It can be noticed that only one promoter variable is present (speed of sound (P)) for response C2. One Slater interaction term (for RAPEX and FWHH (M)) and a quadratic term (for rAPEX (M)) can be seen in the table. Brinell hardness (M) and electron affinity (M) seem to be also strong variables in addition to temperature.

From Table S12, it can be seen that two promoter variables are chosen (dummy variable for Cr (P) and first ionization energy (P) for response S2). Slater interaction or quadratic terms are not seen. Volume magnetic susceptibility (M), Brinell hardness (M), and electronegativity (M) are the most often occurring variables after temperature.

As seen in Table S13, temperature and boiling point (M) occur in every subset with yield Y1. Also, density (M) Brinell hardness (M), bulk modulus (M), and electron affinity (M) seem to be strong variables for predicting Y1. In addition, the presence of main metals Pt, Ir, and Pd has a great impact on the yield results with diethyl carbonate solvent according to the chosen subsets. Promoter variables or STO variables cannot be found from the table of the most influential descriptors.

From Table S14, it can be seen that in addition to temperature, Brinell hardness occurs in almost every subset. Also, the interaction term between RAPEX and FWHH (M), electron affinity (M), first ionization energy (M), and neutron cross section (M) seem to be strong descriptors for predicting yield with 1,4-dioxane solvent (Y2). The presence of main metals Ir and Pd seem to be most influential according to the chosen variable subsets (as this can be also noticed from high experimental yield values). Once again, promoter variables cannot be found from the table.

From the results for the occurrence of variables, the following conclusions can be made: (1) promoter variables seem to be more relevant in the prediction of selectivity than conversion. (2) STO variables and their interactions and quadratic terms seem to be more relevant for predicting conversion than selectivity. (3) Temperature was present in every variable subset with all responses. Also, several descriptors were found important, including Brinell hardness (M), electron affinity (M), and Slater interaction term for RAPEX and FWHH (M). (4) In general, main metal variables are much more relevant than promoter variables, which is also the case with the studied datasets. Analysis of variance (ANOVA) shows that the variation is clearly due to main metal variables (mean squares > 1300 for all temperatures for dataset C1) while promoter variables seem to have less effect on the variation (mean squares < 80). The *p*-values from the ANOVA also show that the group means based on promoter metals do not have a statistically significant difference (*p*-values > 0.3). (5) It is also notable that the fitrlinear function rarely chooses dummy variables in the variable subset. Therefore, the chosen variable subsets with fitrlinear function and lasso function differ significantly.

For comparison, the modeling was also performed with the two most important variables identified based on variable selection results (Section 3.2), namely, the temperature and Brinell hardness (M). The results can be seen in Table S7 in the last rows for each response variable (cf. variable subset VII). The RMSEP values are only slightly worse or even better for responses C1, S1, and Y1 than with the variable subsets chosen by variable selection algorithms. As discussed earlier, the Brinell hardness was proven to be a strong descriptor. With the studied dataset, these two variables explain most of the variance in the response data. It was surprising that with the responses, which had low correlation (S1, Y1, and Y2) against Brinell hardness and temperature, good modeling results were still obtained.

## 4 Conclusions

The application of ML in catalyst development has shown great promise. In this work, a systematic approach for testing different variable selection algorithms and model structures was considered for modeling catalyst performance (conversion, selectivity, and yield). For the studied case of hydrogenation of 5-ethoxymethylfurfural with simple bimetal catalysts, it was shown that relatively high modeling accuracy can be achieved (correlation varying between 0.90 and 0.98) through the utilization of regularization algorithms, RLA models, and descriptor dataset of STO parameters with the addition of variables found in the literature.

The importance of systematic variable selection is supported by the results as it seems to have a beneficial impact on the models’ performance. It was also shown that fairly good results can be obtained with only two input variables in this case. Brinell hardness for main metal was found to have high predictive power. Promoter variables were considered unimportant with variable selection algorithms that can be an issue when deriving optimal catalyst formulations, where both the main metal and promoter need to be selected.

In general, the best results were obtained with GPR, SVR, and fine decision tree methods. From the studied variable selection algorithms, different model structures perform best with different responses. Even though the modeling results were good, the variable selection methods were almost purely data-driven, and the physical interpretation of all of the variables remains unclear. Also, some of the values in the descriptor dataset were obtained from compiled lists from multiple experimental and simulated studies. Therefore, these are likely to contain a certain amount of inaccuracy (for example, uncertainties in atomic radius or in the measurement of other physical properties). The lasso algorithm was introduced with datasets consisting of highly correlated variables, which can lead to the algorithm picking one variable and ignoring the remaining ones, resulting in loss of potentially significant variables (see Section 2.3). Despite that fact, with test set, good results were obtained for five responses with correlation ranging between 0.89 and 0.97.

In the future work, model-based optimization is to be studied with the goal of finding catalysts that give the maximum FOM values. Also, the model extrapolation capabilities could be further studied. Moreover, also other relevant descriptors can be identified and added to the dataset (for example, d-band center values).

## Supporting Information Available

Detailed modeling results; variable subsets for the best models; variable occurrences in variable selection; full list of the used descriptors; details regarding the data division (PDF)
Descriptor data file (XLSX)
