# Supplementary material for: Systematic Data-Driven Modeling of Bimetallic Catalyst Performance for the Hydrogenation of 5-Ethoxymethylfurfural with Variable Selection and Regularization
Source: Ind Eng Chem Res. 2022 Mar 31;61(14):4752–62. doi: 10.1021/acs.iecr.1c03995 (PMC9014324; doi:10.1021/acs.iecr.1c03995)
Supplement: Supplementary file 1 — ie1c03995_si_001.pdf [file ie1c03995_si_001.pdf]

Supporting Information

Systematic data-driven modelling of bimetallic catalyst performance for hydrogenation of 5-ethoxymethylfurfural with variable selection and regularization

Pekka Uusitalo[a*], Aki Sorsa[a], Fernando Russo Abegão[b], Markku Ohenoja[a], Mika Ruusunen[a]

[a]Environmental and Chemical Engineering Research Unit, Control Engineering Group, Faculty of Technology, P.O.Box 4300, University of Oulu, Oulu, 90014, Finland

[b]School of Engineering, Newcastle University, Newcastle upon Tyne, NE1 7RU, United Kingdom

*Email: pekka.uusitalo@oulu.fi

Table S1. Root mean square error values, correlations and prediction uncertainties for reference models of conversion with diethyl carbonate solvent (C1). RMSET = Root mean squared value for training set. RMSECV = Root mean squared value of cross-validation. RMSEP = Root mean squared value for test set. RT = Correlation between observed and predicted values with training set. RCV = Correlation between observed and predicted values with cross-validation set. RP = Correlation between observed and predicted values with test set. PUT = Prediction Uncertainty (mean value of error ± 2 * standard deviation of error) with training set. PUCV = Prediction Uncertainty with cross-validation set. PUP = Prediction Uncertainty with test set.

**Model No.**	**Modelling Method**	**RMSET (%)**	**RMSECV mean/std (%)**	**RMSEP (%)**	**RT**	**RCV**	**RP**	**PUT (e ± 2σ) (%)**	**PUCV (e ± 2σ) (%)**	**PUP (e ± 2σ) (%)**
**1.**	Ridge Regression (`fitrlinear`)	10.58	12.74 / 0.65	13.96	0.86	0.83	0.77	0.33 +- 21.26	-0.45 +- 23.42	-0.4 +- 28.2
**2.**	Lasso Regression (`fitrlinear`)	10.10	12.44 / 0.57	13.96	0.88	0.82	0.77	-0.02 +- 20.3	-0.4 +- 24.16	-0.74 +- 28.18
**3.**	Lasso Regression (`lasso`)	9.77	9.46 / 0.41	13.75	0.88	0.83	0.78	0 +- 19.64	0.03 +- 23.17	-0.72 +- 27.75
**4.**	Elastic Net (`lasso`)	9.77	9.52 / 0.46	13.75	0.88	0.84	0.78	0 +- 19.64	0.09 +- 23.01	-0.72 +- 27.75
**5.**	PLSR	9.81	11.80 / 0.39	13.72	0.88	0.83	0.78	0 +- 19.73	0.08 +- 23.23	-0.72 +- 27.69

Table S2. Root mean square error values, correlations and prediction uncertainties for reference models of selectivity with diethyl carbonate solvent (S1).

**Model No.**	**Modelling Method**	**RMSET (%)**	**RMSECV mean/std (%)**	**RMSEP (%)**	**RT**	**RCV**	**RP**	**PUT (e ± 2σ) (%)**	**PUCV (e ± 2σ) (%)**	**PUP (e ± 2σ) (%)**
**6.**	Ridge Regression (`fitrlinear`)	15.07	15.50 / 0.62	16.18	0.69	0.72	0.65	2.73 +- 29.8	2.57 +- 29.47	3.05 +- 32.13
**7.**	Lasso Regression (`fitrlinear`)	13.88	15.57 / 0.51	14.99	0.80	0.71	0.76	2.38 +- 27.5	2.94 +- 30.3	2.7 +- 29.81
**8.**	Lasso Regression (`lasso`)	11.73	11.38 / 0.48	14.69	0.82	0.73	0.72	0 +- 23.58	0.15 +- 27.98	0.32 +- 29.7

**9.**	Elastic Net (`lasso`)	11.73	11.51 / 0.46	14.69	0.82	0.74	0.72	0 +- 23.58	0.05 +- 27.77	0.32 +- 29.69
**10.**	PLSR	11.82	14.49 / 0.60	15.23	0.81	0.72	0.70	0 +- 23.77	0.12 +- 28.36	0.32 +- 30.78

Table S3. Root mean square error values, correlations and prediction uncertainties for reference models of conversion with 1,4-dioxane solvent (C2).

**Model No.**	**Modelling Method**	**RMSET (%)**	**RMSECV mean/std (%)**	**RMSEP (%)**	**RT**	**RCV**	**RP**	**PUT (e ± 2σ) (%)**	**PUCV (e ± 2σ) (%)**	**PUP (e ± 2σ) (%)**
**11.**	Ridge Regression (`fitrlinear`)	13.33	14.78 / 0.69	16.76	0.91	0.90	0.85	0.84 +- 26.75	1.56 +- 27.99	-0.27 +- 33.88
**12.**	Lasso Regression (`fitrlinear`)	12.20	14.63 / 0.64	15.72	0.92	0.89	0.87	1.55 +- 24.32	1.41 +- 28.57	0.44 +- 31.76
**13.**	Lasso Regression (`lasso`)	11.09	10.77 / 0.51	15.62	0.93	0.91	0.87	0 +- 22.3	0.01 +- 26.21	-1.11 +- 31.49
**14.**	Elastic Net (`lasso`)	11.09	10.79 / 0.69	15.61	0.93	0.91	0.87	0 +- 22.3	0.01 +- 26.56	-1.11 +- 31.48
**15.**	PLSR	11.28	14.30 / 0.84	15.94	0.93	0.90	0.86	0 +- 22.68	-0.02 +- 27.66	-1.11 +- 32.15

Table S4. Root mean square error values, correlations and prediction uncertainties for reference models of selectivity with 1,4-dioxane solvent (S2).

**Model No.**	**Modelling Method**	**RMSET (%)**	**RMSECV mean/std (%)**	**RMSEP (%)**	**RT**	**RCV**	**RP**	**PUT (e ± 2σ) (%)**	**PUCV (e ± 2σ) (%)**	**PUP (e ± 2σ) (%)**
**16.**	Ridge Regression (`fitrlinear`)	34.07	40.35 / 1.93	38.61	0.64	0.49	0.50	-6.35 +- 67.3	-6.14 +- 77.23	-1.98 +- 77.95
**17.**	Lasso Regression (`fitrlinear`)	33.68	39.98 / 1.77	37.56	0.65	0.51	0.54	-3.39 +- 67.37	-3.8 +- 77.37	0.98 +- 75.9
**18.**	Lasso Regression (`lasso`)	31.98	31.13 / 1.11	39.44	0.68	0.53	0.48	0 +- 64.29	-0.02 +- 75.53	4.37 +- 79.23

**19.**	Elastic Net (`lasso`)	31.98	31.23 / 1.25	39.43	0.68	0.54	0.48	0 +- 64.29	0.04 +- 75.04	4.37 +- 79.22
**20.**	PLSR	32.08	38.27 / 1.34	39.09	0.68	0.53	0.49	0 +- 64.49	0.42 +- 75.34	4.37 +- 78.52

Table S5. Root mean square error values, correlations and prediction uncertainties for reference models of yield with diethyl carbonate solvent (Y1).

**Model No.**	**Modelling Method**	**RMSET (%)**	**RMSECV mean/std (%)**	**RMSEP (%)**	**RT**	**RCV**	**RP**	**PUT (e ± 2σ) (%)**	**PUCV (e ± 2σ) (%)**	**PUP (e ± 2σ) (%)**
**21.**	Ridge Regression (`fitrlinear`)	13.59	14.46 / 0.52	15.65	0.65	0.62	0.57	2.72 +- 26.77	2.97 +- 27.3	2.71 +- 31.17
**22.**	Lasso Regression (`fitrlinear`)	13.93	14.86 / 0.27	15.51	0.75	0.63	0.73	3.64 +- 27.03	3.79 +- 28.73	3.64 +- 30.48
**23.**	Lasso Regression (`lasso`)	10.44	10.20 / 0.41	13.95	0.78	0.68	0.65	0 +- 21	0.02 +- 24.82	0 +- 28.2
**24.**	Elastic Net (`lasso`)	10.44	10.32 / 0.37	13.95	0.78	0.68	0.65	0 +- 21	0 +- 24.78	0 +- 28.19
**25.**	PLSR	10.51	12.90 / 0.53	14.34	0.78	0.67	0.63	0 +- 21.13	0 +- 25.29	0 +- 28.99

Table S6. Root mean square error values, correlations and prediction uncertainties for reference models of yield with 1,4-dioxane solvent (Y2).

**Model No.**	**Modelling Method**	**RMSET (%)**	**RMSECV mean/std (%)**	**RMSEP (%)**	**RT**	**RCV**	**RP**	**PUT (e ± 2σ) (%)**	**PUCV (e ± 2σ) (%)**	**PUP (e ± 2σ) (%)**
**26.**	Ridge Regression (`fitrlinear`)	15.95	18.93 / 1.32	18.74	0.75	0.66	0.65	-2.02 +- 31.81	1.88 +- 36.18	-3.2 +- 37.34
**27.**	Lasso Regression (`fitrlinear`)	16.37	19.95 / 0.84	17.83	0.75	0.61	0.69	1.55 +- 32.77	3.38 +- 38.58	0.37 +- 36.04
**28.**	Lasso Regression (`lasso`)	15.10	14.85 / 0.95	18.50	0.78	0.68	0.66	0 +- 30.36	0.01 +- 35.72	-1.18 +- 37.32

**29.**	Elastic Net (`lasso`)	15.10	14.59 / 1.04	18.50	0.78	0.67	0.66	0 +- 30.36	-0.05 +- 35.97	-1.18 +- 37.32
**30.**	PLSR	15.12	18.26 / 0.90	18.62	0.78	0.67	0.65	0 +- 30.4	0.04 +- 35.92	-1.18 +- 37.57

Table S7. The best modelling results (18 models) according to RMSEP values for six different responses.

**Model No.**	**Response**	**Variable Selection Method**	**Model structure**	**RMSET** (%)	**RMSECV mean/std** (%)	**RMSEP (%)**	**RT**	**RCV**	**RP**	**PUT (e ± 2σ) (%)**	**PUCV (e ± 2σ) (%)**	**PUP (e ± 2σ) (%)**	**Number of Variables**	**Variable subset**
**31.**	C1	-	Quadratic SVM	4.05	11.02	9.51	0.98	0.91	0.90	-0.11 +- 8.14	-0.35 +- 17.44	-1.31 +- 19.05	147	-
**32.**	C1	-	Exponential GPR	6.16	8.52	9.60	0.96	0.92	0.90	0 +- 12.39	0.02 +- 16.38	-0.96 +- 19.32	2	I.
**33.**	C1	Ridge (fitrlinear)	Cubic SVM	6.49	8.72	9.61	0.95	0.92	0.90	-0.31 +- 13.04	-0.45 +- 16.19	-1.26 +- 19.26	6	VII.
**34.**	S1	-	Fine Tree	5.11	9.28	5.24	0.97	0.92	0.97	0 +- 10.28	0 +- 16.05	0.27 +- 10.57	147	-
**35.**	S1	Ridge (fitrlinear)	Fine Tree	5.31	7.43	5.12	0.97	0.94	0.97	0 +- 10.67	0.07 +- 13.99	0.3 +- 10.33	7	II.
**36.**	S1	-	Matern 5/2 GPR	5.13	7.46	4.87	0.97	0.94	0.97	0 +- 10.31	0.12 +- 13.72	0.01 +- 9.84	2	VII.
**37.**	Y1	-	Fine Tree	3.27	5.90	5.01	0.98	0.96	0.96	0 +- 6.57	0.31 +- 9.25	-0,51 +- 10,08	147	-
**38.**	Y1	Ridge (fitrlinear)	Cubic SVM	3.34	4.76	4.05	0.98	0.97	0.98	0.42 +- 6.67	0.29 +- 8.11	0.04 +- 8.2	7	III.
**39.**	Y1	-	Rational Quadratic GPR	3.04	4.79	4.22	0.98	0.97	0.97	0 +- 6.12	0.09 +- 8.44	-0.35 +- 8.5	2	VII.
**40.**	C2	-	Quadratic SVM	4.50	13.82	9.05	0.99	0.94	0.96	0.12 +- 9.04	-0.37 +- 20.9	-1.35 +- 18.09	147	-
**41.**	C2	Lasso (fitrlinear)	Matern 5/2 GPR	3.51	9.91	9.09	0.99	0.95	0.96	0 +- 7.06	0.09 +- 18.67	-0.75 +- 18.31	13	IV.
**42.**	C2	-	Squared Exponential GPR	6.59	9.30	10.24	0.98	0.96	0.95	0 +- 13.26	0.14 +- 17.57	-1.32 +- 20.53	2	VII.
**43.**	S2	-	Boosted Ensemble Tree	18.02	34.15	34.51	0.92	0.67	0.66	2.31 +- 35.93	2.6 +- 65.53	10.05 +- 66.75	147	-
**44.**	S2	Ridge (fitrlinear)	Fine Tree	24.95	34.21	34.12	0.82	0.70	0.65	0 +- 50.18	-0.68 +- 62.32	4.37 +- 68.41	10	V.
**45.**	S2	-	Fine Tree	25.45	34.90	34.10	0.81	0.67	0.65	0 +- 51.16	-0.53 +- 65.45	4.37 +- 68.36	2	VII.
**46.**	Y2	-	Quadratic SVM	9.23	18.88	13.09	0.93	0.78	0.84	1.41 +- 18.33	-0.17 +- 31.06	-0.02 +- 26.46	147	-
**47.**	Y2	Ridge (fitrlinear)	Cubic SVM	6.07	7.81	8.75	0.97	0.95	0.94	0.75 +- 12.1	0.39 +- 14.55	-0.7 +- 17.63	8	VI.
**48.**	Y2	-	Squared Exponential GPR	5.56	7.88	9.27	0.97	0.95	0.93	0 +- 11.17	0 +- 14.87	-1.38 +- 18.52	2	VII.

Table S8. Variable subsets for the best modelling results according to RMSEP values. M = main metal, P = promoter, CS = crystal structure, SGN = space group number, IE = ionisation energy, BCM = base-centered monoclinic, FCC = face-centered cubic, SH = simple hexagonal. Definitions for all the variables can be found in tables S15-S19.

**Response**	**Variable subset**	**Variable names**

**C1**	**I**	Temperature, BrinellHardness_M, Resistivity_M, VolumeMagneticSusceptibility_M, LatticeAngle3_M, RAPEX*FWHH_M
**S1**	**II**	Temperature, BoilingPoint_M, HeatofVaporization_M, BulkModulus_M, ValenceofIon_M, ElectronAffinity_M, VolumeMagneticSusceptibility_P
**Y1**	**III**	Temperature, BoilingPoint_M, HeatofVaporization_M, BrinellHardness_M, BulkModulus_M, ValenceofIon_M, ElectronAffinity_M
**C2**	**IV**	Temperature, BrinellHardness_M, ElectronAffinity_M, FirstIE_M, Resistivity_M, NeutronCrossSection_M, rAPEX^2_M, FWHH^2_M, rAPEX*FWHH_M, RAPEX*FWHH_M, ElectronAffinity_P, CovalentRadius_P, LatticeAngle2_P
**S2**	**V**	Temperature, Density_M, BrinellHardness_M, ElectroNegativity_M, FirstIE_M, VolumeMagneticSusceptibility_M, LatticeConstant3_M, NeutronCrossSection_M, RAPEX^2_M, FWHH*SKEW_M
**Y2**	**VI**	Temperature, BrinellHardness_M, BulkModulus_M, ValenceofIon_M, ElectroNegativity_M, ElectronAffinity_M, FirstIE_M, NeutronCrossSection_M
**C1, S1, Y1, C2, S2, Y2**	**VII**	Temperature, BrinellHardness_M

Table S9. Variable occurrences for conversion (C1) with four different regularization methods and their sum. EN = Elastic net with `lasso` function with alpha value 0.5 and λ value corresponding to minimum mean squared error value (minMSE). L1 = Lasso with `lasso` function with minMSE λ value. L2 = lasso with `fitrlinear` function. RR = Ridge with `fitrlinear` function. M = main metal. P = promoter.

	**45.**	**57.**	**93.**	**59.**	**86.**	**71.**	**6.**	**126.**	**89.**
**EN**	20	20	0	0	0	0	20	0	0
**L1**	20	20	20	20	20	0	17	12	0
**L2**	20	20	20	20	20	19	0	20	12
**RR**	20	20	20	0	0	20	0	0	9
**SUM**	80	80	60	40	40	39	37	32	21

45. Temperature, 57. Brinell hardness (M), 93. Interaction term for RAPEX and FWHH (M), 59. Bulk modulus (M), 86. Quadratic term for rAPEX (M), 71. Volume magnetic susceptibility (M), 6. Dummy variable for Pt (M), 126. Second lattice angle (P), 89. Quadratic term for SKEW (M)

Table S10. Variable occurrences for selectivity (S1) with four different regularization methods and their sum.

	**45.**	**50.**	**59.**	**66.**	**48.**	**5.**	**6.**	**11.**	**17.**	**32.**	**122.**	**120.**
**EN**	20	20	20	20	20	20	20	20	20	20	0	0
**L1**	20	20	20	0	20	20	20	20	20	12	11	19
**L2**	20	20	20	20	12	0	0	0	0	0	16	2
**RR**	20	20	20	20	5	3	3	0	0	0	0	0
**SUM**	80	80	80	60	57	43	43	40	40	32	27	21

45. Temperature, 50. Boiling point (M), 59. Bulk modulus (M), 66. Electron affinity (M), 48. Density (M), 5. Dummy variable for Pd (M), 6. Dummy variable for Pt (M), 11. Dummy variable for Fe (P), 17. Dummy variable for group 9 in periodic table (M), 32. Dummy variable for group 8 in periodic table (P), 122. Volume magnetic susceptibility (P), 120. Electrical conductivity (P)

Table S11. Variable occurrences for conversion (C2) with four different regularization methods and their sum.

	**45.**	**57.**	**66.**	**93.**	**1.**	**3.**	**5.**	**67.**	**80.**	**86.**	**114.**
**EN**	20	20	20	20	20	20	20	0	0	0	0
**L1**	20	20	0	20	20	20	20	0	0	20	20
**L2**	20	20	20	20	0	0	0	20	20	19	1
**RR**	20	0	20	0	0	0	0	20	20	0	0
**SUM**	80	60	60	60	40	40	40	40	40	39	21

45. Temperature, 57. Brinell hardness (M), 66. Electron affinity (M), 93. Interaction term for RAPEX and FWHH (M), 1. Dummy variable for Au (M), 3. Dummy variable for Ir (M), 5. Dummy variable for Pd (M), 67. First ionization energy (M), 80. Neutron cross section (M), 86. Quadratic term for rAPEX (M), 114. Speed of sound (P)

Table S12. Variable occurrences for selectivity (S2) with four different regularization methods and their sum.

	**45.**	**71.**	**57.**	**65.**	**1.**	**2.**	**3.**	**4.**	**6.**	**8.**	**10.**	**16.**	**17.**	**79.**	**118.**
**EN**	20	20	20	19	20	20	20	20	20	20	20	20	20	0	0
**L1**	20	7	0	0	20	20	20	20	20	20	20	20	20	0	20
**L2**	20	20	20	20	0	0	0	0	0	0	0	0	0	20	14
**RR**	20	20	20	20	0	0	0	0	0	0	0	0	0	20	0
**SUM**	80	67	60	59	40	40	40	40	40	40	40	40	40	40	34

45. Temperature, 71. Volume magnetic susceptibility (M), 57. Brinell hardness (M), 65. Electronegativity (M), 1. Dummy variable for Au (M), 2. Dummy variable for Cu (M), 3. Dummy variable for Ir (M), 4. Dummy variable for Ni (M), 6. Dummy variable for Pt (M), 8. Dummy variable for Rt (M), 10. Dummy variable for Cr (P), 16. Dummy variable for group 8 in the periodic table (M), 17. Dummy variable for group 9 in the periodic table (M), 79. Third lattice constant (M), 118. First ionization energy (P)

Table S13. Variable occurrences for yield (Y1) with four different regularization methods and their sum.

	**45.**	**50.**	**48.**	**57.**	**59.**	**66.**	**62.**	**6.**	**71.**	**3.**	**17.**	**52.**	**5.**
**EN**	20	20	20	20	20	20	20	20	17	20	20	20	20
**L1**	20	20	20	0	0	0	0	20	2	20	20	0	16
**L2**	20	20	20	20	20	20	20	0	20	0	0	0	0
**RR**	20	20	0	20	20	20	10	5	2	0	0	20	0
**SUM**	80	80	60	60	60	60	50	45	41	40	40	40	36

45. Temperature, 50. Boiling point (M), 48. Density (M), 57. Brinell hardness (M), 59. Bulk modulus (M), 66. Electron affinity (M), 62. Poisson ratio (M), 6. Dummy variable for Pt (M), 71. Volume magnetic susceptibility (M), 3. Dummy variable for Ir (M), 17. Dummy variable for group 9 in the periodic table (M), 52. Heat of vaporization (M), 5. Dummy variable for Pd (M)

Table S14. Variable occurrences for yield (Y2) with four different regularization methods and their sum.

	**45.**	**57.**	**93.**	**66.**	**67.**	**80.**	**3.**	**5.**	**61.**	**65.**
**EN**	20	20	20	20	20	20	20	20	7	8
**L1**	20	20	20	0	0	0	20	20	0	0
**L2**	20	20	20	20	20	20	0	0	19	0
**RR**	20	17	2	20	20	20	5	1	9	20
**SUM**	80	77	62	60	60	60	45	41	35	28

45. Temperature, 57. Brinell hardness (M), 93. Interaction term for RAPEX and FWHH (M), 66. Electron affinity (M), 67. First ionization energy (M), 80. Neutron cross section (M), 3. Dummy variable for Ir (M), 5. Dummy variable for Pd (M), 61. Young modulus (M), 65. Electronegativity (M)

Table S15. Variables used in the variable selection, part 1. M refers to main metal and P to promoter.

Number	Variable	Unit	Definition
**1.**	Dummy variable for Au (M)	N/A	Explains the presence of Au as main metal.
**2.**	Dummy variable for Cu (M)	N/A	Explains the presence of Cu as main metal.
**3.**	Dummy variable for Ir (M)	N/A	Explains the presence of Ir as main metal.
**4.**	Dummy variable for Ni (M)	N/A	Explains the presence of Ni as main metal.
**5.**	Dummy variable for Pd (M)	N/A	Explains the presence of Pd as main metal.
**6.**	Dummy variable for Pt (M)	N/A	Explains the presence of Pt as main metal.
**7.**	Dummy variable for Rh (M)	N/A	Explains the presence of Rh as main metal.
**8.**	Dummy variable for Rt (M)	N/A	Explains the presence of Rt as main metal.
**9.**	Dummy variable for Bi (P)	N/A	Explains the presence of Bi as promoter.
**10.**	Dummy variable for Cr (P)	N/A	Explains the presence of Cr as promoter.
**11.**	Dummy variable for Fe (P)	N/A	Explains the presence of Fe as promoter.
**12.**	Dummy variable for Na (P)	N/A	Explains the presence of Na as promoter.
**13.**	Dummy variable for Sn (P)	N/A	Explains the presence of Sn as promoter.
**14.**	Dummy variable for W (P)	N/A	Explains the presence of W as promoter.
**15.**	Dummy variable for d-block in the periodic table (M)	N/A	Explains, if the main metal belongs to d-block in the periodic table.
**16.**	Dummy variable for group 8 in the periodic table (M)	N/A	Explains, if the main metal belongs to group 8 in the periodic table.
**17.**	Dummy variable for group 9 in the periodic table (M)	N/A	Explains, if the main metal belongs to group 9 in the periodic table.
**18.**	Dummy variable for group 10 in the periodic table (M)	N/A	Explains, if the main metal belongs to group 10 in the periodic table.
**19.**	Dummy variable for group 11 in the periodic table (M)	N/A	Explains, if the main metal belongs to group 11 in the periodic table.
**20.**	Dummy variable for period 4 in the periodic table (M)	N/A	Explains, if the main metal belongs to period 4 in the periodic table.
**21.**	Dummy variable for period 5 in the periodic table (M)	N/A	Explains, if the main metal belongs to period 5 in the periodic table.
**22.**	Dummy variable for period 6 in the periodic table (M)	N/A	Explains, if the main metal belongs to period 6 in the periodic table.
**23.**	Dummy variable for face-centered cubic crystalline structure (M)	N/A	Explains, if the main metal has face-centered cubic crystalline structure.
**24.**	Dummy variable for simple hexagonal crystalline structure (M)	N/A	Explains, if the main metal has simple hexagonal crystalline structure.
**25.**	Dummy variable for space group number 194 (M)	N/A	Explains, if the main metal has space group number 194.
**26.**	Dummy variable for space group number 225 (M)	N/A	Explains, if the main metal has space group number 194.
**27.**	Dummy variable for d-block in the periodic table (P)	N/A	Explains, if the promoter belongs to d-block in the periodic table.
**28.**	Dummy variable for p-block in the periodic table (P)	N/A	Explains, if the promoter belongs to p-block in the periodic table.
**29.**	Dummy variable for s-block in the periodic table (P)	N/A	Explains, if the promoter belongs to s-block in the periodic table.
**30.**	Dummy variable for group 1 in the periodic table (P)	N/A	Explains, if the promoter belongs to group 1 in the periodic table.
**31.**	Dummy variable for group 6 in the periodic table (P)	N/A	Explains, if the promoter belongs to group 6 in the periodic table.

Table S16. Variables used in the variable selection, part 2. M refers to main metal and P to promoter.

**Number**	**Variable**	**Unit**	**Definition**
**32.**	Dummy variable for group 8 in the periodic table (P)	N/A	Explains, if the promoter belongs to group 8 in the periodic table.
**33.**	Dummy variable for group 14 in the periodic table (P)	N/A	Explains, if the promoter belongs to group 14 in the periodic table.
**34.**	Dummy variable for group 15 in the periodic table (P)	N/A	Explains, if the promoter belongs to group 15 in the periodic table.
**35.**	Dummy variable for period 3 in the periodic table (P)	N/A	Explains, if the promoter belongs to period 3 in the periodic table.
**36.**	Dummy variable for period 4 in the periodic table (P)	N/A	Explains, if the promoter belongs to period 4 in the periodic table.
**37.**	Dummy variable for period 5 in the periodic table (P)	N/A	Explains, if the promoter belongs to period 5 in the periodic table.
**38.**	Dummy variable for period 6 in the periodic table (P)	N/A	Explains, if the promoter belongs to period 6 in the periodic table.
**39.**	Dummy variable for base-centered monoclinic crystalline structure (P)	N/A	Explains, if the promoter has base-centered monoclinic crystalline structure.
**40.**	Dummy variable for base-centered cubic crystalline structure (P)	N/A	Explains, if the promoter has base-centered cubic crystalline structure.
**41.**	Dummy variable for centered tetragonal crystalline structure (P)	N/A	Explains, if the promoter has centered tetragonal crystalline structure.
**42.**	Dummy variable for space group number 12 (P)	N/A	Explains, if the promoter has space group number 12.
**43.**	Dummy variable for space group number 141 (P)	N/A	Explains, if the promoter has space group number 141.
**44.**	Dummy variable for space group number 229 (P)	N/A	Explains, if the promoter has space group number 229.
**45.**	Temperature	°C	Temperature of experiment.
**46.**	Atomic number (M)	N/A	Atomic number of the element in periodic table.
**47.**	Atomic weight (M)	g/mol	Defines the weight of an atom.
**48.**	Density (M)	g/cm^3	Defines materials mass per unit volume.
**49.**	Melting point (M)	K	Defines the temperature value at which the element changes its phase from solid to liquid.
**50.**	Boiling point (M)	K	Defines the temperature value at which the element changes its phase from liquid to gas.
**51.**	Heat of fusion (M)	kJ/mol	The quantity of heat necessary to change a solid to a liquid without temperature change.
**52.**	Heat of vaporization (M)	kJ/mol	The quantity of heat necessary to change a liquid to a solid without temperature change.
**53.**	Specific heat capacity (M)	J/(kg*K)	The quantity of heat necessary for a given mass to produce a unit change in its temperature.
**54.**	Thermal conductivity (M)	W/(m*K)	A measure of materials ability to conduct heat.
**55.**	Thermal expansion (M)	K^{-1}	Defines materials ability to change its shape, area, volume, and density to a temperature change.
**56.**	Molar volume (M)	m^3/mol	Volume occupied by one mole of the substance at the given temperature and pressure.
**57.**	Brinell hardness (M)	MPa	Definition of materials hardness tested by applying pressure with indenter on the material.
**58.**	Mohs hardness (M)	N/A	Defines materials scratch resistance.
**59.**	Bulk modulus (M)	GPa	Defines materials resistance to compression.
**60.**	Shear modulus (M)	GPa	Describe materials response to shear stress.

Table S17. Variables used in the variable selection, part 3. M refers to main metal and P to promoter.

Number	Variable	Unit	Definition
**61.**	Young modulus (M)	GPa	Defines materials resistance to elastic changes.
**62.**	Poisson ratio (M)	N/A	A measure of the Poisson effect.
**63.**	Speed of sound (M)	m/s	Defines how fast sound will travel in the material.
**64.**	Valence of ion (M)	N/A	Defines the number of electrons in the materials valence orbital.
**65.**	Electronegativity (M)	N/A	Defines atoms ability to attract a shared pair of electrons with another.
**66.**	Electron affinity (M)	kJ/mol	Defines the change in energy of a neutral atom, when an electron is added to the atom to form a negative ion.
**67.**	First ionization energy (M)	kJ/mol	The amount of energy needed to remove one electron from an atom.
**68.**	Second ionization energy (M)	kJ/mol	The amount of energy needed to remove two electrons from an atom.
**69.**	Electrical conductivity (M)	S/m	Defines materials ability to conduct electric current.
**70.**	Resistivity (M)	m*Ω	Defines materials ability to resist electric current.
**71.**	Volume magnetic susceptibility (M)	N/A	Indicates the degree of magnetization of a material in response to an applied magnetic field.
**72.**	Atomic radius (M)	pm	Measure of the size of atoms in element.
**73.**	Covalent radius (M)	pm	Measure of the size of atom that forms part of one covalent bond.
**74.**	First lattice angle (M)	N/A	Defines first dimension’s angle in unit cell that describes the crystal structure.
**75.**	Second lattice angle (M)	N/A	Defines second dimension’s angle in unit cell that describes the crystal structure.
**76.**	Third lattice angle (M)	N/A	Defines third dimension’s angle in unit cell that describes the crystal structure.
**77.**	First lattice constant (M)	pm	Defines first dimension’s length in unit cell that describes the crystal structure.
**78.**	Second lattice constant (M)	pm	Defines second dimension’s length in unit cell that describes the crystal structure.
**79.**	Third lattice constant (M)	pm	Defines third dimension’s length in unit cell that describes the crystal structure.
**80.**	Neutron cross section (M)	b	Defines the likelihood of interaction between an incident neutron and a target nucleus.
**81.**	Neutron mass absorption (M)	m^2/kg	Thermal neutron mass absorption coefficient.
**82.**	STO variable rAPEX (M)	N/A	Distance of maximum probability of encountering a valence electron.
**83.**	STO variable RAPEX (M)	N/A	Maximum value of the probability distribution (i.e. STOs).
**84.**	STO variable FWHH (M)	N/A	Width of the probability distribution (i.e. STOs) at half height (half of the maximum).
**85.**	STO variable SKEW (M)	N/A	Measure for the asymmetry of the probability distribution (i.e. STOs).
**86.**	Quadratic term for rAPEX (M)	N/A	Quadratic term for rAPEX.
**87.**	Quadratic term for RAPEX (M)	N/A	Quadratic term for RAPEX.
**88.**	Quadratic term for FWHH (M)	N/A	Quadratic term for FWHH.
**89.**	Quadratic term for SKEW (M)	N/A	Quadratic term for SKEW.

Table S18. Variables used in the variable selection, part 4. M refers to main metal and P to promoter.

Number	Variable	Unit	Definition
**90.**	Interaction term for rAPEX and RAPEX (M)	N/A	Interaction term for rAPEX and RAPEX.
**91.**	Interaction term for rAPEX and FWHH (M)	N/A	Interaction term for rAPEX and FWHH.
**92.**	Interaction term for rAPEX and SKEW (M)	N/A	Interaction term for rAPEX and SKEW.
**93.**	Interaction term for RAPEX and FWHH (M)	N/A	Interaction term for RAPEX and FWHH.
**94.**	Interaction term for RAPEX and SKEW (M)	N/A	Interaction term for RAPEX and SKEW.
**95.**	Interaction term for FWHH and SKEW (M)	N/A	Interaction term for FWHH and SKEW.
**96.**	Surface energy (M)	J/m	Defines the surface excess free energy per unit area of a particular crystal facet.
**97.**	Atomic number (P)	N/A	Atomic number of the element in periodic table.
**98.**	Atomic weight (P)	g/mol	Defines the weight of an atom.
**99.**	Density (P)	g/cm^3	Defines materials mass per unit volume.
**100.**	Melting point (P)	K	Defines the temperature value at which the element changes its phase from solid to liquid.
**101.**	Boiling point (P)	K	Defines the temperature value at which the element changes its phase from liquid to gas.
**102.**	Heat of fusion (P)	kJ/mol	The quantity of heat necessary to change a solid to a liquid without temperature change.
**103.**	Heat of vaporization (P)	kJ/mol	The quantity of heat necessary to change a liquid to a solid without temperature change.
**104.**	Specific heat capacity (P)	J/(kg*K)	The quantity of heat necessary for a given mass to produce a unit change in its temperature.
**105.**	Thermal conductivity (P)	W/(m*K)	A measure of materials ability to conduct heat.
**106.**	Thermal expansion (P)	K-1	Defines materials ability to change its shape, area, volume, and density to a temperature change.
**107.**	Molar volume (P)	m3/mol	Volume occupied by one mole of the substance at the given temperature and pressure.
**108.**	Brinell hardness (P)	MPa	Definition of materials hardness tested by applying pressure with indenter on the material.
**109.**	Mohs hardness (P)	N/A	Defines materials scratch resistance.
**110.**	Bulk modulus (P)	GPa	Defines materials resistance to compression.
**111.**	Shear modulus (P)	GPa	Describe materials response to shear stress.
**112.**	Young modulus (P)	GPa	Defines materials resistance to elastic changes.
**113.**	Poisson ratio (P)	N/A	A measure of the Poisson effect.
**114.**	Speed of sound (P)	m/s	Defines how fast sound will travel in the material.
**115.**	Valence of ion (P)	N/A	Defines the number of electrons in the materials valence orbital.
**116.**	Electronegativity (P)	N/A	Defines atoms ability to attract a shared pair of electrons with another.
**117.**	Electron affinity (P)	kJ/mol	Defines the change in energy of a neutral atom, when an electron is added to the atom to form a negative ion.
**118.**	First ionization energy (P)	kJ/mol	The amount of energy needed to remove one electron from an atom.
**119.**	Second ionization energy (P)	kJ/mol	The amount of energy needed to remove two electrons from an atom.
**120.**	Electrical conductivity (P)	S/m	Defines materials ability to conduct electric current.
**121.**	Resistivity (P)	m*Ω	Defines materials ability to resist electric current.

Table S19. Variables used in the variable selection, part 5. M refers to main metal and P to promoter.

Number	Variable	Unit	Definition
**122.**	Volume magnetic susceptibility (P)	N/A	Indicates the degree of magnetization of a material in response to an applied magnetic field.
**123.**	Atomic radius (P)	pm	Measure of the size of atoms in element.
**124.**	Covalent radius (P)	pm	Measure of the size of atom that forms part of one covalent bond.
**125.**	First lattice angle (P)	N/A	Defines first dimension's angle in unit cell that describes the crystal structure.
**126.**	Second lattice angle (P)	N/A	Defines second dimension's angle in unit cell that describes the crystal structure.
**127.**	Third lattice angle (P)	N/A	Defines third dimension's angle in unit cell that describes the crystal structure.
**128.**	First lattice constant (P)	pm	Defines first dimension's length in unit cell that describes the crystal structure.
**129.**	Second lattice constant (P)	pm	Defines second dimension's length in unit cell that describes the crystal structure.
**130.**	Third lattice constant (P)	pm	Defines third dimension's length in unit cell that describes the crystal structure.
**131.**	Neutron cross section (P)	b	Defines the likelihood of interaction between an incident neutron and a target nucleus.
**132.**	Neutron mass absorption (P)	m2/kg	Thermal neutron mass absorption coefficient.
**133.**	Slater variable rAPEX (P)	N/A	Distance of maximum probability of encountering a valence electron.
**134.**	Slater variable RAPEX (P)	N/A	Maximum value of the probability distribution (i.e. STOs).
**135.**	Slater variable FWHH (P)	N/A	Width of the probability distribution (i.e. STOs) at half height (half of the maximum).
**136.**	Slater variable SKEW (P)	N/A	Measure for the asymmetry of the probability distribution (i.e. STOs).
**137.**	Quadratic term for rAPEX (P)	N/A	Quadratic term for rAPEX.
**138.**	Quadratic term for RAPEX (P)	N/A	Quadratic term for RAPEX.
**139.**	Quadratic term for FWHH (P)	N/A	Quadratic term for FWHH.
**140.**	Quadratic term for SKEW (P)	N/A	Quadratic term for SKEW.
**141.**	Interaction term for rAPEX and RAPEX (P)	N/A	Interaction term for rAPEX and RAPEX.
**142.**	Interaction term for rAPEX and FWHH (P)	N/A	Interaction term for rAPEX and FWHH.
**143.**	Interaction term for rAPEX and SKEW (P)	N/A	Interaction term for rAPEX and SKEW.
**144.**	Interaction term for RAPEX and FWHH (P)	N/A	Interaction term for RAPEX and FWHH.
**145.**	Interaction term for RAPEX and SKEW (P)	N/A	Interaction term for RAPEX and SKEW.
**146.**	Interaction term for FWHH and SKEW (P)	N/A	Interaction term for FWHH and SKEW.
**147.**	Surface energy (P)	J/m	Defines the surface excess free energy per unit area of a particular crystal facet.

Table S20. Data division, part 1.

Index	Dataset (Train/Test)	Main metal	Promoter	Temperature (°C)
**1**	Test	Au	Bi	80
**2**	Train	Au	Bi	100
**3**	Train	Au	Bi	120
**4**	Train	Au	Cr	80
**5**	Test	Au	Cr	100
**6**	Train	Au	Cr	120
**7**	Train	Au	Fe	80
**8**	Train	Au	Fe	100
**9**	Test	Au	Fe	120
**10**	Test	Au	Na	80
**11**	Train	Au	Na	100
**12**	Train	Au	Na	120
**13**	Train	Au	Sn	80
**14**	Test	Au	Sn	100
**15**	Train	Au	Sn	120
**16**	Train	Au	W	80
**17**	Train	Au	W	100
**18**	Test	Au	W	120
**19**	Train	Cu	Bi	80
**20**	Test	Cu	Bi	100
**21**	Train	Cu	Bi	120
**22**	Train	Cu	Cr	80
**23**	Train	Cu	Cr	100
**24**	Test	Cu	Cr	120
**25**	Test	Cu	Fe	80
**26**	Train	Cu	Fe	100
**27**	Train	Cu	Fe	120
**28**	Train	Cu	Na	80
**29**	Test	Cu	Na	100
**30**	Train	Cu	Na	120
**31**	Train	Cu	Sn	80
**32**	Train	Cu	Sn	100
**33**	Test	Cu	Sn	120
**34**	Test	Cu	W	80
**35**	Train	Cu	W	100
**36**	Train	Cu	W	120
**37**	Train	Ir	Bi	80
**38**	Train	Ir	Bi	100
**39**	Test	Ir	Bi	120
**40**	Test	Ir	Cr	80
**41**	Train	Ir	Cr	100
**42**	Train	Ir	Cr	120

Table S21. Data division, part 2.

Index	Dataset (Train/Test)	Main metal	Promoter	Temperature (°C)
**43**	Train	Ir	Fe	80
**44**	Test	Ir	Fe	100
**45**	Train	Ir	Fe	120
**46**	Train	Ir	Na	80
**47**	Train	Ir	Na	100
**48**	Test	Ir	Na	120
**49**	Test	Ir	Sn	80
**50**	Train	Ir	Sn	100
**51**	Train	Ir	Sn	120
**52**	Test	Ni	Bi	80
**53**	Train	Ni	Bi	100
**54**	Train	Ni	Bi	120
**55**	Train	Ni	Cr	80
**56**	Test	Ni	Cr	100
**57**	Train	Ni	Cr	120
**58**	Train	Ni	Fe	80
**59**	Train	Ni	Fe	100
**60**	Test	Ni	Fe	120
**61**	Test	Ni	Na	80
**62**	Train	Ni	Na	100
**63**	Train	Ni	Na	120
**64**	Train	Ni	Sn	80
**65**	Test	Ni	Sn	100
**66**	Train	Ni	Sn	120
**67**	Train	Ni	W	80
**68**	Train	Ni	W	100
**69**	Test	Ni	W	120
**70**	Train	Pd	Bi	80
**71**	Test	Pd	Bi	100
**72**	Train	Pd	Bi	120
**73**	Train	Pd	Cr	80
**74**	Train	Pd	Cr	100
**75**	Test	Pd	Cr	120
**76**	Test	Pd	Fe	80
**77**	Train	Pd	Fe	100
**78**	Train	Pd	Fe	120
**79**	Train	Pd	Na	80
**80**	Test	Pd	Na	100
**81**	Train	Pd	Na	120
**82**	Train	Pd	Sn	80
**83**	Train	Pd	Sn	100
**84**	Test	Pd	Sn	120

Table S22. Data division, part 3.

Index	Dataset (Train/Test)	Main metal	Promoter	Temperature (°C)
**85**	Test	Pd	W	80
**86**	Train	Pd	W	100
**87**	Train	Pd	W	120
**88**	Train	Pt	Bi	80
**89**	Train	Pt	Bi	100
**90**	Test	Pt	Bi	120
**91**	Test	Pt	Cr	80
**92**	Train	Pt	Cr	100
**93**	Train	Pt	Cr	120
**94**	Train	Pt	Fe	80
**95**	Test	Pt	Fe	100
**96**	Train	Pt	Fe	120
**97**	Train	Pt	Na	80
**98**	Train	Pt	Na	100
**99**	Test	Pt	Na	120
**100**	Test	Pt	Sn	80
**101**	Train	Pt	Sn	100
**102**	Train	Pt	Sn	120
**103**	Train	Pt	W	80
**104**	Test	Pt	W	100
**105**	Train	Pt	W	120
**106**	Test	Rh	Bi	80
**107**	Train	Rh	Bi	100
**108**	Train	Rh	Bi	120
**109**	Train	Rh	Cr	80
**110**	Test	Rh	Cr	100
**111**	Train	Rh	Cr	120
**112**	Train	Rh	Fe	80
**113**	Train	Rh	Fe	100
**114**	Test	Rh	Fe	120
**115**	Test	Rh	Na	80
**116**	Train	Rh	Na	100
**117**	Train	Rh	Na	120
**118**	Train	Rh	Sn	80
**119**	Test	Rh	Sn	100
**120**	Train	Rh	Sn	120
**121**	Train	Rh	W	80
**122**	Train	Rh	W	100
**123**	Test	Rh	W	120
**124**	Train	Ru	Bi	80
**125**	Test	Ru	Bi	100
**126**	Train	Ru	Bi	120

Table S23. Data division, part 4.

Index	Dataset (Train/Test)	Main metal	Promoter	Temperature (°C)
**127**	Train	Ru	Cr	80
**128**	Train	Ru	Cr	100
**129**	Test	Ru	Cr	120
**130**	Test	Ru	Fe	80
**131**	Train	Ru	Fe	100
**132**	Train	Ru	Fe	120
**133**	Train	Ru	Na	80
**134**	Test	Ru	Na	100
**135**	Train	Ru	Na	120
**136**	Train	Ru	Sn	80
**137**	Train	Ru	Sn	100
**138**	Test	Ru	Sn	120
**139**	Test	Ru	W	80
**140**	Train	Ru	W	100
**141**	Train	Ru	W	120
